# Proteomic Approaches Identify Members of Cofilin Pathway Involved in Oral Tumorigenesis

**DOI:** 10.1371/journal.pone.0050517

**Published:** 2012-12-05

**Authors:** Giovana M. Polachini, Lays M. Sobral, Ana M. C. Mercante, Adriana F. Paes-Leme, Flávia C. A. Xavier, Tiago Henrique, Douglas M. Guimarães, Alessandra Vidotto, Erica E. Fukuyama, José F. Góis-Filho, Patricia M. Cury, Otávio A. Curioni, Pedro Michaluart Jr, Adriana M. A. Silva, Victor Wünsch-Filho, Fabio D. Nunes, Andréia M. Leopoldino, Eloiza H. Tajara

**Affiliations:** 1 Departamento de Biologia Molecular; Faculdade de Medicina (FAMERP), São José do Rio Preto, SP, Brazil; 2 Departamento de Análises Clínicas, Toxicológicas e Bromatológicas, Faculdade de Ciências Farmacêuticas da Universidade de São Paulo, Ribeirão Preto, SP, Brazil; 3 Laboratório de Patologia, Hospital Heliópolis, São Paulo, SP, Brazil; 4 Laboratório Nacional de Biociências (LNBio), Centro Nacional de Pesquisa em Energia e Materiais, Campinas, SP, Brazil; 5 Departamento de Propedêutica e Clínica Integrada, Faculdade de Odontologia da Universidade Federal da Bahia, Salvador,BA, Brazil; 6 Departamento de Estomatologia, Faculdade de Odontologia da Universidade de São Paulo, São Paulo, SP, Brazil; 7 Serviço de Cirurgia de Cabeça e Pescoço, Instituto do Câncer Arnaldo Vieira de Carvalho, São Paulo, SP, Brazil; 8 Departamento de Patologia e Medicina Legal, Faculdade de Medicina (FAMERP), São José do Rio Preto, SP, Brazil; 9 Departamento de Cirurgia de Cabeça e Pescoço e Otorrinolaringologia, Hospital Heliópolis, São Paulo, SP, Brazil; 10 Divisão de Cirurgia de Cabeça e Pescoço, Departamento de Cirurgia, Faculdade de Medicina da Universidade de São Paulo, São Paulo, SP, Brazil; 11 Departamento de Produção Vegetal, Universidade Federal do Espírito Santo, Vitória, ES, Brazil; 12 Departamento de Epidemiologia, Faculdade de Saúde Pública da Universidade de São Paulo, São Paulo, SP, Brazil; 13 Departamento de Genética e Biologia Evolutiva, Instituto de Biociências da Universidade de São Paulo, São Paulo, SP, Brazil; Thomas Jefferson University, United States of America

## Abstract

The prediction of tumor behavior for patients with oral carcinomas remains a challenge for clinicians. The presence of lymph node metastasis is the most important prognostic factor but it is limited in predicting local relapse or survival. This highlights the need for identifying biomarkers that may effectively contribute to prediction of recurrence and tumor spread. In this study, we used one- and two-dimensional gel electrophoresis, mass spectrometry and immunodetection methods to analyze protein expression in oral squamous cell carcinomas. Using a refinement for classifying oral carcinomas in regard to prognosis, we analyzed small but lymph node metastasis-positive versus large, lymph node metastasis-negative tumors in order to contribute to the molecular characterization of subgroups with risk of dissemination. Specific protein patterns favoring metastasis were observed in the “more-aggressive” group defined by the present study. This group displayed upregulation of proteins involved in migration, adhesion, angiogenesis, cell cycle regulation, anti-apoptosis and epithelial to mesenchymal transition, whereas the “less-aggressive” group was engaged in keratinocyte differentiation, epidermis development, inflammation and immune response. Besides the identification of several proteins not yet described as deregulated in oral carcinomas, the present study demonstrated for the first time the role of cofilin-1 in modulating cell invasion in oral carcinomas.

## Introduction

Similar to other head and neck tumors, oral squamous cell carcinomas (OSCCs) are heterogeneous with regard to anatomic subsites, clinical presentation and outcome. They affect the mucosa between the lip and the palate including oral tongue, floor of the mouth, buccal mucosa, alveolar ridge and retromolar trigone (International Classification of diseases/ICD, http://www.who.int/classifications/icd/en/). Clinically, OSCCs may remain locally invasive or be more aggressive and metastasize. Actually, histologically similar lesions can follow significantly different clinical courses and show different responses to therapy, probably as a result of etiologic and molecular heterogeneity [1], as well as of microenvironmental factors [2], [3].

Most oral carcinomas metastasize to regional lymph nodes instead of spreading to distant sites via hematogeneous routes. The anatomic subsite and its microenvironment, including secreted lymphangiogenic growth factors and lymphatic microvessel density, may influence this dissemination pattern [4]–[6]. However, to invade adjacent vessels, the tumor cells also need to change their plasticity, from an epithelial to a more mesenchymal phenotype [7], [8], consequently with decreased epithelial adhesion, remodeling of cytoskeleton and increased migratory features. Finally, the success of the process depends on the survival of the neoplastic cells in the vessels, escape from immune response, extravasation and proliferation in lymph nodes [8].

OSCCs are frequently diagnosed at a late stage of the disease, when the chances of cure are low and the treatments are more invasive. Unfortunately, even when the patients present with early-stage disease, recurrence, local and distant metastasis and a second primary tumor can occur. To predict the outcome of these patients, TNM (tumor, node, metastasis) staging of the tumor, especially the presence of regional lymph node metastasis, is still the most important factor [9]. However, the clinical evaluation of lymph node spread and relapse risks in patients with nonmetastatic disease mainly depend on the tumor diameter and subsite. Because the prognostic significance of these parameters is limited [10], many histopathological markers have been investigated in the recent decades: some are of little value in prognostication and others show more consistent associations [11]. Several examples may be mentioned, such as tumor thickness [12], histological grading [13], apoptotic indices [14], DNA ploidy [15], microvascular density [16], [17] and vascular and perineural invasion [18].

Following the advances in high-throughput technologies, numerous studies aiming to investigate molecular markers able to predict behavior of OSCC have been published in the recent years. Their results have shown a large number of gene and protein expression patterns associated with progression and outcome of head and neck cancer [1], [19]. Cellular pathways modulated by these proteins are frequently related to the cancer hallmarks described by Hanahan and Weinberg [20], [21], and linked to proliferation, apoptosis, energy metabolism, immune evasion, angiogenesis, invasion and metastasis processes. However, most of these studies are based on a small sample size, which limits the use of their data in clinical practice [11]. Particularly for prognosticators of lymph node and distant metastasis, the literature data are limited [1] but recent findings have suggested that certain expression profiles can predict tumor dissemination or be related to epithelial-to-mesenchymal transition [8], [22].

In the present study, we used two-dimensional gel electrophoresis (2-DE), mass spectrometry (MS) and immunodetection methods to analyze protein expression in OSCCs and their surgical margins. To maximize the coverage of proteins, we also performed one-dimensional gel electrophoresis (1-DE) followed by MS identification. Using a refinement for classifying oral carcinomas in regard to prognosis, we analyzed small but already lymph node metastasis-positive (N+) versus large, lymph node metastasis-negative (N0) tumors. The rationale for this exploratory classification is our limited ability to predict tumor behavior in patients with OSCC based on only the presence of regional lymph node metastasis. The purpose of the study is to enrich the molecular characterization of OSCC subgroups with different prognoses, especially those with risk of tumor dissemination.

## Materials and Methods

### Ethics Statement

The study protocol and the informed consent were approved by the Committees on Ethics in Research of Heliopolis Hospital, Arnaldo Vieira de Carvalho Cancer Institute, Clinics Hospital of the Faculty of Medicine (University of São Paulo) and by the National Committee on Ethics in Research/CONEP (reference number 1763/05, 18/05/2005). All patients provided their written consent to participate in the study after being informed about the research purposes.

### Patients and Specimens

Two hundred fifty-two samples of primary OSCC and surgical margin were obtained from a total of 144 patients. None of the patients had received preoperative radiation or chemotherapy. The samples were collected by the Head and Neck Genome Project (GENCAPO), a collaborative consortium of research groups from hospitals and universities in São Paulo State, Brazil, whose aim is to develop clinical, genetic and epidemiological analysis of head and neck squamous cell carcinomas.

Immediately after surgery, the specimen was cut in two: one part was snap-frozen and stored in liquid nitrogen and the other part was fixed in formalin for routine histopathological examination. Analysis of hematoxylin and eosin-stained sections indicated that each tumor sample contained at least 70% tumor cells and the surgical margins were “tumor-free”. The total set of samples was derived from four oral subsites (C02 =  other and unspecified parts of tongue; C03 =  gum; C04 =  floor of mouth; C06 =  other and unspecified parts of mouth), according to the criteria established by the World Health Organization (WHO) (http://apps.who.int/classifications/apps/icd/icd10online/). Proteomic experiments were carried out on samples from C02 and C04 subsites and immunodetection analysis on samples from all subsites.

The tumors were classified by the TNM system [23]. Using a refinement for classifying oral carcinomas in regard to prognosis, small but lymph node metastasis-positive tumors (T1-2N+) at diagnosis were considered potentially “more-aggressive” (MA), and large, lymph node metastasis-negative ones (T2-3N0) were considered “less-aggressive” (LA). A full description of the clinicopathological data and techniques used to analyze the samples is provided in Table S1.

### Protein Extraction

Since the macrodissected clinical samples were small (from 0.3 cm^3^ to 0.5 cm^3^) and different fractions should be obtained from the same sample for analysis on various platforms in a multicenter study, proteins were extracted after RNA extraction by TRIzol® Reagent (Invitrogen Corp., Carlsbad, CA, USA). Briefly, the organic phase containing DNA and proteins was isolated, DNA was precipitated with ethanol and, subsequently, the proteins of the supernatant fraction were precipitated with isopropyl alcohol. The pellets were then maintained for 10 min at 15 to 30°C, sedimented at 12,000 g for 10 min at 4°C and washed three times with 0.3 M guanidine hydrochloride in 95% ethanol. During each wash step, the pellets were maintained in the washing solution for 20 min at room temperature and centrifuged at 7,500 g for 5 min at 4°C. After the last wash, the pellets were vortexed in ethanol, stored for 20 min at room temperature and centrifuged at 7,500 g for 5 min at 4°C. The samples were dried for 5 to 10 min and diluted in 1% sodium dodecyl sulphate (SDS) at 50°C. After centrifugation at 10,000 g for 10 min at 4°C, the supernatants were recovered and protein quantification was performed using the detergent-compatible BCA™ Protein Assay Kit (Pierce Biotechnology, Rockford, IL, USA). All protein samples were stored in aliquots at −80°C until analysis.

To minimize individual differences and to enable multiple analyses of samples with a limited amount of proteins, one- and two-dimensional gel electrophoresis were performed using pooled samples grouped according to anatomic subsites and TNM system. In total, 51 individual samples (28 tumors and 23 surgical margins) from smoking patients older than 40 years were combined in pools of tumors or surgical margins.

**Figure 1:**
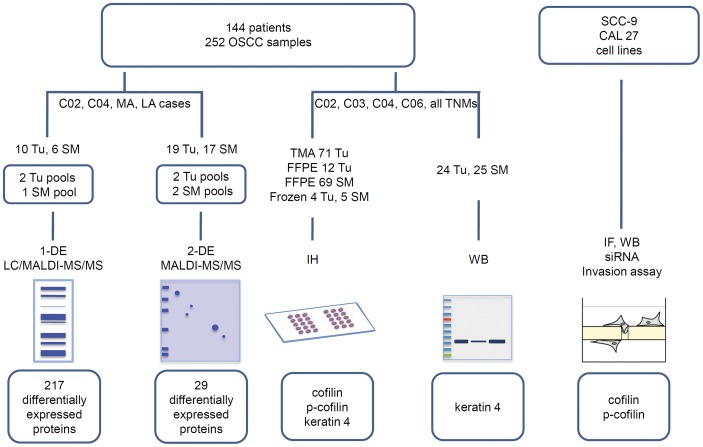
Flowchart of sample preparation and analysis. Tu = tumor; SM = surgical margin; MA or more-aggressive = T1-2N+; LA or less-aggressive = T2-3N0; TMA = tissue microarray; FFPE = formalin-fixed, paraffin-embedded; 1-DE = one-dimensional gel electrophoresis; 2-DE = two-dimensional gel electrophoresis; LC = liquid chromatography; MALDI = matrix assisted laser desorption ionization; MS/MS = tandem mass spectrometry; WB = Western blot; IH = immunohistochemistry; IF = immunofluorescence; siRNA = small interfering RNA.

The pools were prepared by mixing equal amounts of protein from each sample, resulting in a total of 100 and 1500 µg per pool for 1-DE and 2-DE gels, respectively. A description of all the pools is presented in Table S2. The flowchart of sample preparations and analysis is shown in Figure 1.

### One-dimensional Gel Electrophoresis (1-DE)

Protein pools of small N+ tumors and of large N0 tumors, and one protein pool of their surgical margins were separated in one-dimensional 12% resolving/5% stacking SDS-polyacrylamide gel (SDS-PAGE). In total, 16 samples (10 tumor and 6 surgical margin samples from 11 patients) were analyzed.

Briefly, the proteins were denatured at 98°C for 10 min in 5X loading buffer with dithiothreitol (DTT) and 100 µg of each pool were loaded into the wells. SDS-PAGE was carried out in a vertical polyacrylamide gel system (SE 400 Vertical Unit, GE Healthcare, Uppsala, Sweden) at 130 V. Molecular mass was estimated using low molecular weight standard proteins of 14.4–97 kDa (LMW Calibration Kit for SDS Electrophoresis, GE Healthcare). Proteins were detected by Coomassie Brilliant Blue staining. Each gel lane (from 14.4 to 97 kDa) was cut into 21 slices of approximately equal size. The slices were destained, dehydrated and digested with trypsin (Promega Corp., Madison, WI, USA).

### Two-dimensional Gel Electrophoresis (2-DE)

In a similar way, protein pools of small N+ tumors and of large N0 tumors, and protein pools of their surgical margins were analyzed by 2-DE according to the protocol previously described by de Marqui et al. [24] *with modifications*. In total, 36 samples (19 tumor and 17 surgical margin samples from 23 patients) were analyzed.

Proteins were precipitated using ice-cold 100% acetone and resuspended in rehydration solution (8 M urea, 2% w/v CHAPS detergent, 0.3% w/v DTT, 0.5% v/v IPG buffer pH 3–10, bromophenol blue trace) to a final volume of 250 µL before loaded onto immobilized 13 cm linear pH gradient (IPG) strips (pH 3–10, GE Healthcare). Isoelectric focusing (IEF) was performed for a total of 26,500 Vh at 20°C and 50 µA/strip, using an Ettan IPGphor Isoelectric Focusing (GE Healthcare).

IPG strips were equilibrated for 15 min in equilibration solution [6 M urea, 50 mM Tris-HCl pH 8.8, 30% v/v glycerol (87% v/v), 2% w/v SDS, bromophenol blue trace] containing 1% w/v DTT, followed by incubation for 15 min in the same solution containing 2.5% w/v iodoacetamide instead of DTT. IPG strips were sealed on top of 12.5% SDS-polyacrylamide gels using 0.5% w/v low-melting agarose in SDS running buffer with bromophenol blue. Electrophoresis was performed at 15 mA per gel for 30 min at room temperature, followed by 30 mA per gel for 3–5 h, in a Hoefer SE 600 Ruby system (GE Healthcare). All samples were run in duplicate or triplicate to guarantee reproducibility. The LMW Calibration Kit was used as a protein standard.

Gels were Coomassie Blue-stained, scanned using an ImageScanner (GE Healthcare) and the images were analyzed using the ImageMaster 2D Platinum software, version 6.0 (GE Healthcare) for spot detection, quantification, and comparative and statistical analysis. Basically, tumor and surgical margin groups or tumor stage groups were created and the gels were matched to a reference gel. The spot quantification for each gel was based on the relative volume (percent volume), i.e. the volume of each spot divided by the total volume over the whole image. Differential analysis of images was performed by matching spots from two gels of each group (“more-aggressive” and “less-aggressive” tumors and their surgical margins). The data obtained were evaluated statistically using the Student’s *t*-test. Statistical significance was set at *p*<0.05.

### Mass Spectrometry (MS), Protein Identification and Annotation

Bands from 1-DE gel were excised, reduced, alkylated and submitted to in-gel digestion with trypsin [25]. An aliquot (4.5 µL) of the resulting peptide mixture was separated by C18 (100 µm×100 mm) RP-nanoUPLC (nanoAcquity, Waters Corp., Milford, MA, USA) coupled with a Q-Tof Ultima mass spectrometer (Waters) with nano-electrospray source at a flow rate of 0.6 µL/min. The gradient was 2–90% acetonitrile (ACN) in 0.1% formic acid over 45 min. The instrument was operated in the “top three” mode, in which one MS spectrum is acquired followed by MS/MS of the top three most-intense peaks detected. The spectra were acquired using the MassLynx software version 4.1 (Waters) and the raw data files were converted to a peak list format (mgf) by the Mascot Distiller software, version 2.2.1.0, 2008 (Matrix Science Ltd., London, UK).

The search results of 1-D gel electrophoresis were exported to Scaffold Q+ software (version 3_00_03, Proteome Software Inc., Portland, OR, USA) and visualized using a filter as described by Escalante et al. [26] and Eming et al. [27]. The protein identification probability was >95%, one minimum peptide, and 95% of peptide probability. Briefly, proteins from the 21 gel slices from each pool were grouped in three categories: more-aggressive or less-aggressive tumors or surgical margins. As proposed by Escalante et al. [26], relative quantification was obtained by the quantitative value, which normalizes the spectral counts across the experiment. This value represents an average total of nongrouped spectral counts for a protein divided by the total nongrouping spectral counts for the mass spectral runs from the gel slices from each lane, which allows for a relative quantitative comparison of a specific protein among samples. The normalized spectral counts were obtained for each protein and the proteins with a fold change of at least 1.5 were considered with differential abundance between the categories. Fold change was calculated using the statistical tests of Scaffold Q+ software (according to [28]).

Differentially expressed protein spots from 2-DE gels were excised and also digested with trypsin [24]. Peptide digest were mixed with matrix solution (10 mg/mL α-cyano-4-hydroxycinnamic acid, 0.1% v/v trifluoroacetic acid/TFA in 50% v/v ACN) in a 1∶1 (v:v) ratio, spotted on a stainless steel sample plate and air dried. Mass determinations were performed on a MALDI TOF-TOF (Matrix Assisted Laser Desorption Ionization - Time of Flight - Time of Flight) 4700 Proteomics Analyzer (Applied Biosystems, Foster City, CA, USA) or a MALDI Q-TOF (Matrix Assisted Laser Desorption Ionization - Quadrupole Ion Trap - Time of Flight) Premier (Waters). Each sample was run in triplicate. For protein identification, the data were searched against a non-redundant protein *Homo sapiens* database (NCBI nr 2009.07.20, 9,298,190 sequences) using a MASCOT engine version 2.0 (Matrix Science Ltd.), with carbamidomethylation of cysteine as fixed modification, oxidation of methionine as variable modification, one trypsin missed cleavage and a tolerance of 1 or 0.1 Da for both precursor and fragment ions.

Gene Ontology (GO) annotation (http://www.geneontology.org/) was used to assign biological process terms for differentially expressed proteins. To analyze biological functions, proteins with altered expression profiles were imported into Ingenuity Pathway Analysis Tool (IPA Tool, Ingenuity®Systems, Redwood City, CA, USA).

### Validation Experiments

Complementary methodologies were performed to validate the proteomic findings. The selection of proteins for validation experiments was carried out after an extensive literature analysis. The following criteria were used: (i) potential involvement in cancer development or a yet unclear role in OSCC tumorigenesis, (ii) expression pattern suggestive of a role in aggressive OSSC behavior. Using these criteria as guidelines, one protein overexpressed in tumors and related to an invasive phenotype was selected (cofilin-1). A known marker of head and neck squamous cell carcinomas with a pronounced underexpression in tumors and previously associated with malignant progression (keratin 4) was also selected to validate our strategy.

#### Western blot

A subset of 49 samples (24 tumors and 25 surgical margins) from 30 patients was analyzed by Western blot. The antibodies used were mouse monoclonal antibody anti-keratin 4 (6B10, sc-52321, Santa Cruz Biotechnology, Santa Cruz, CA, USA) diluted 1∶500, mouse monoclonal anti-α-tubulin (TU-02, sc-8035, Santa Cruz Biotechnology) diluted 1∶500 and mouse monoclonal anti-β-actin (AC-15, A1978, Sigma-Aldrich, Saint Louis, MO, USA) diluted 1∶5000. Protein samples (9 µg) were separated by SDS-PAGE (12% resolving gel with 5% stacking gel) at 130 V for 60 min (Mini-Protean 3 Cell Electrophoresis System, BioRad, Hercules, CA, USA), in denaturing conditions. The molecular weight ladders used were the PageRuler™ Prestained Protein Ladder (SM0671, Fermentas Life Sciences, Burlington, ON, Canada) and the See Blue Plus2 Pre-Stained Standard (Invitrogen).

The proteins were transferred (325 mA for 70 min) to polyvinylidene difluoride/PVDF membranes (Immobilon-P, Millipore Corp., Billerica, MA, USA) using transfer buffer (25 mM Tris, 0.2 M glycine, 20% v/v methanol) and the Mini Trans-Blot Electrophoretic Transfer Cell (BioRad). The membranes were submitted to chromogenic staining using the Western Breeze Immunodetection Kit (Invitrogen), according to the manufacturer’s protocol. The blots were analyzed using Gel Logic HP 2200 imaging system (Carestream Health Inc/Kodak Health Group, Rochester, NY, USA).

#### Immunohistochemistry

For immunohistochemical analysis, two tissue microarray (TMA) slides from 71 primary OSCC samples, 12 tissue slides containing archival formalin-fixed, paraffin-embedded tissue (FFPE) sections of OSCC samples, 69 tissue slides containing FFPE sections of paired surgical margins, and frozen sections from 5 cases (4 tumors and 5 surgical margins) were used (Table S1). In TMA, two representative tumor areas of each case were selected from a hematoxylin- and eosin-stained section of a donor block. Two cylinders per patient (diameter of 1 mm each) were punched out and arrayed in a recipient paraffin block using an arraying device (Beecher Instruments, Silver Spring, MD, USA). Therefore, each tissue microarray contained 142 cores of tumor samples.

Cofilin-1 and keratin 4 immunohistochemical analyses of TMA or FFPE samples was performed by a pathologist using conventional protocols. Briefly, after deparaffinization in xylene and rehydration in graded ethanol, antigen epitope retrieval was performed using 10 mM citrate buffer, pH 6.0 in a vapor cooker. Endogenous peroxidase activity was blocked with 3% hydrogen peroxide for 15 min. Rabbit monoclonal anti-cofilin (D3F9-XP, 5175, Cell Signaling, Boston, MA, USA), rabbit polyclonal anti-cofilin (phospho S3) (ab12866, Abcam, Cambridge, UK) diluted 1∶400 and 1∶300, respectively, and mouse monoclonal antibody anti-keratin 4 (sc-52321, Santa Cruz Biotechnology) diluted 1∶200, were incubated overnight at 8°C followed by addition of the secondary antibody and streptavidin-biotin peroxidase (LSAB+, K0690, Dako, Carpinteria, CA, USA) or performed according to standard protocols using the Advance™ HRP Detection kit (K4068, Dako). Color of reaction product was developed by 3,3′-diaminobenzidine (DAB, Dako) and counterstaining was performed with Harris hematoxylin.

The primary antibody was omitted for negative controls. The extent of immunoreactivity (R) for cofilin-1 was semiquantitatively graded as 0 (<10% of immunoreactive cells), score 1 (10–25% of immunoreactive cells), score 2 (25–50% of immunoreactive cells), score 3 (>50% of immunoreactive cells). For keratin 4, R was graded as 0 (<5%), score 1 (5–10%), score 2 (11–50%), score 3 (51–75%) and score 4 (>75%). The staining intensity was evaluated as negative (0), mild (1), moderate (2) and intense (3).

Cofilin-1 immunohistochemical analyses of frozen tissue samples were also performed using the streptavidin-biotin peroxidase complex method. Tissue sections from stored frozen samples were first stained with toluidine blue and reanalyzed to confirm the presence of tumor cells or normal epithelium. Five-µm-thick cryostat sections were then mounted on silane-coated glass slides, immersed in cold methanol for 15 min, rinsed 3 times with phosphate-buffered saline (PBS) for 2 min, followed by the treatment with 3% hydrogen peroxide. The slides were treated with a solution of 1% bovine serum albumin (BSA) in PBS for 1 h and then incubated for 16 h at 4°C with either rabbit monoclonal anti-cofilin (D3F9-XP, 5175, Cell Signaling) diluted 1∶200 or with rabbit polyclonal anti-cofilin (phospho S3) (ab12866, Abcam) diluted 1∶200. Subsequent incubations were performed with the secondary antibody and streptavidin-biotin peroxidase (LSAB+, K0690, Dako), developed with DAB and counterstained with Mayer hematoxylin.

#### Immunofluorescence

The SCC-9 cell line (ATCC, Manassas, VA, USA), which is derived from a squamous cell carcinoma of the tongue, was grown in a 1∶1 mixture of Dulbecco’s Modified Eagle’s Medium (DMEM, Invitrogen) and Ham’s F12 Medium (DMEM/F12, Invitrogen) supplemented with 10% fetal bovine serum (FBS), 400 ng/ml hydrocortisone (Sigma-Aldrich) and antibiotic/antimycotic (Sigma-Aldrich). After 24 h, the cells were fixed with cold methanol for 15 min at room temperature and rinsed 3 times with PBS for 2 min. Cells were then treated with a solution of 1% BSA in PBS for 1 h and incubated for 1 h at 25°C with rabbit polyclonal anti-cofilin (phospho S3) (ab12866; Abcam) diluted 1∶100, rinsed 3 times with PBS and then incubated for 1 h at 25°C with the secondary antibody donkey anti-rabbit IgG conjugated to Alexa Fluor 488 (A21206, Invitrogen) diluted 1∶1000 for 1 h at 25°C. Nuclei were stained with 4′,6-diamidino-2-phenylindol (DAPI). Coverslips were mounted with fluorescent mounting medium (S3023, Dako).

#### Invasion assay

The effect of cofilin-1 on tumor cell invasion was determined by gene silencing with interference RNA (siRNA) and Boyden chamber assay. The assay was carried out using the SCC-9 and CAL 27 (ATCC) cell lines, the latter derived from a poorly differentiated squamous cell carcinoma at the middle of the tongue.

The cells were seeded in a 12-well plate (1×10^5^ cells/well): SCC-9 cells were cultured overnight in 1∶1 mixture of DMEM and DMEM/F12 and CAL 27 cells were cultured overnight in DMEM. Both media were supplemented with 10% FBS, 400 ng/ml hydrocortisone and antibiotic/antimycotic.

Three siRNA duplex sequences against cofilin-1 (NM_005507, siCofilin I: 187697918, II: 87697919 and III: 87697920, 20 nM) and a negative control siRNA (Integrated DNA Technologies, Coralville, IA, USA) were transfected into cells using Hyperfect reagent (Qiagen, Germantown, MD, USA), according to the manufacturer’s protocol. Knockdown efficiency was confirmed on the protein level using Western blot analysis and immunofluorescence staining. The antibodies used were rabbit monoclonal anti-cofilin (D3F9-XP, 5175, Cell Signaling) diluted 1∶1000, rabbit polyclonal anti-cofilin (phospho S3) (ab12866, Abcam) diluted 1∶1000 and mouse monoclonal anti-α-tubulin (sc-8035, Santa Cruz Biotechnology) diluted 1∶500.

Invasion assay was performed using a Boyden chamber with a polycarbonate filter (8 µm pore size) coated with Matrigel (10 µg/ml). The cells (1×10^5^ suspended in 0.5 mL of serum-free DMEM/F12) were seeded into the upper chamber. The bottom chamber contained medium with 10% FBS. After 72 h of incubation, the nonmigrated cells on the upper surface of the filter were carefully removed. Cells that migrated to the bottom side of the filter were fixed, stained and counted in 10 fields per well at ×200 magnification. The experiments were repeated three times.

## Results

### Patient Characteristics

Of the 144 patients with oral carcinomas included in the present study, 119 (82.6%) were male and 25 (17.4%) female, most were older than 50 years (56.1±10.1 years), with a history of alcohol (84.7%) and tobacco (93.1%) abuse. Eighty-two (56.9%) had N+ and 62 (43.1%) had N0 carcinomas: 22.2% (32/144) of tumors were classified as “more-aggressive” (T1-2N+), 32% (46/144) as “less-aggressive” (T2-3N0), and 45.8% (66/144) as other groups (Table S1).

### Association with Tumor Development

The 1-DE results validated by Scaffold software allowed the identification of 217 differentially expressed proteins (≥1.5-fold change) between tumors and surgical margins, with over 95% confidence (as per the Scaffold algorithm, at least one unique peptide per protein). Using these parameters the false discovery rate (FDR) for protein identification was 0.2% (Table S3). The 2-DE analysis revealed 29 differentially expressed proteins between tumor and margin samples (Student’s t test p<0.05), most also found by 1-DE approach (except actin cytoplasmic 2, carbonic anhydrase 3, myoglobin, myosin light chain 3, superoxide dismutase [Cu-Zn] and troponin T slow skeletal muscle), and with an identical or similar expression pattern. Observed and estimated molecular weight (MW) and isoelectric point (pI) were similar, reinforcing the validity of the results (Table S4, Figures S1–S2).

In summary, after the removal of redundancy, both approaches led to the identification of 223 differentially expressed proteins. Among the overexpressed proteins in cancer samples, many are involved in glycolysis (*α*–enolase, glucose-6-phosphate isomerase and triosephosphate isomerase 1), cell proliferation (*40S ribosomal protein S9*, tenascin), anti-apoptosis (cathepsin D, cofilin-1, heat shock protein β1, stratifin, voltage-dependent anion-selective channel protein 1), signaling (*14-3-3 protein β/α, 40S ribosomal protein S6, collagen α-1 I, g*alectin-1, heat shock protein β1, *Ras-related protein Rap-1A,* stratifin), adhesion (*galectin-1,* periostin, Ras-related protein Rap-1A), migration (collagen α-1 I, myosin 9, prelamin-A/C), cytoskeleton organization (desmin, myosin 9, profilin-1), angiogenesis (ATP synthase β, myosin-9), immune response or inflammation (60 kDa heat shock protein, glutathione S-transferase P, protein S100-A9), and response to stimulus (endoplasmin, profilin-1).

Differential expression was also observed for proteins involved in keratinocyte or epithelial cell differentiation and epidermis development. Overexpression of desmoplakin, stratifin and keratins typical of proliferative basal cells (keratins 5 and 14) and keratinizing oral mucosal epithelia (keratin 6A and 16) was detected in tumor samples, whereas surgical margins exhibited consistently high levels of a keratin characteristic of suprabasal layers of oral nonkeratinizing epithelia (keratin 4).

### Association with Aggressive Phenotype

Patients with “more-aggressive” (MA) and “less-aggressive” (LA) tumors showed a similar gender, age ratio and tumor subsite distribution. The “more-aggressive” group exhibited a tendency toward a younger age at diagnosis, but nonsignificantly (Chi-square  =  2.4576; p = 0.1170).

With respect to the protein profiles, MA and LA tumors revealed fewer differences (but not less important) than *were found between* tumor and surgical margins: 133 differentially expressed proteins were identified by 1-DE (Table S3) and 12 by 2-DE (*Student’s t test p<0.05;* Table S4, Figure S3).

The “less-aggressive” group expressed higher levels of proteins involved in keratinocyte differentiation/epidermis development (cystatin-A, desmoplakin, keratin 9, nucleoside diphosphate kinase B and protein S100-A7) and inflammation/immune response (high mobility group protein B1 and protein S100-A7). As expected, the “more-aggressive” group showed a distinct pattern, with higher expression of proteins involved in metastasis-related processes, including migration (myosin-9), adhesion (α-actinin-1 and periostin), angiogenesis (ATP synthase β and myosin-9), cell cycle (heat shock protein HSP90-α, septin-2, stathmin), anti-apoptosis (60 kDa heat shock protein) and positive regulation of epithelial to mesenchymal transition (collagen α-1(XII) chain).

Remarkable differences between MA and LA tumors were observed in relation to proteins associated with cytoskeleton organization (desmin, keratin 8, septin-2) and cofilin pathway (actin-related protein 2/3 subunit 2 or ARP2/3, adenylyl cyclase-associated protein 1 or CAP1, cofilin-1, F-actin-capping protein α-1 or CAPZA1, HSP 90-α, tropomyosin α-3 isoform 4).

### Validation *of* Upregulation of Cofilin-1 and Downregulation of Keratin 4 by Immunodetection Assays

**Figure 2:**
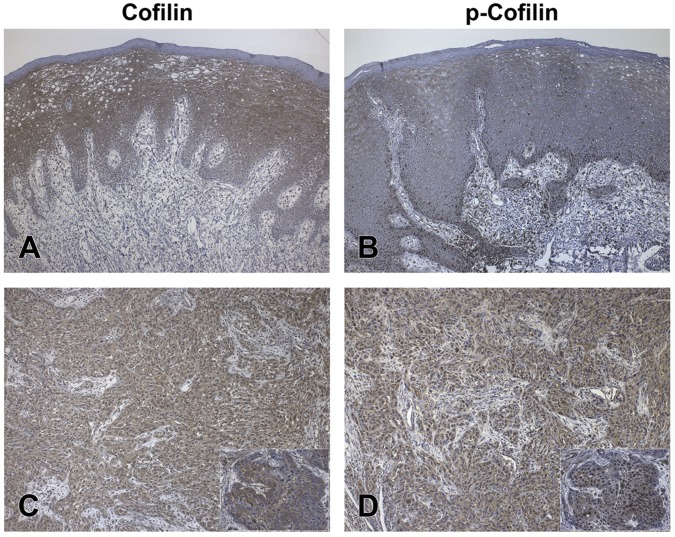
Immunodetection *of* cofilin-1 and p-cofilin in OSCC samples. Immunostaining for total cofilin-1 and p-cofilin in FFPE sections of (A and B, respectively) surgical margins and (C and D, respectively) OSCC samples. Note the low positivity of total cofilin (A) and the nuclear staining for p-cofilin (B) in the more basal layers of epithelium in margins, and (C and D, inserts) the more intense staining of tumor cell nuclei for p-cofilin than for total cofilin. Figures and inserts = 100X and 400X magnification, respectively.

Immunoreactivity for total cofilin-1, an actin regulatory protein essential for directed cell migration in many cell types, was high (80/81) in tumor samples (independently of TNM) and mostly localized in the cytoplasm (Figure 2C), whereas phospho-cofilin (p-cofilin) staining was decreased or negative (7/11) and localized in both nuclei and cytoplasm (Figure 2D). In surgical margins, the intensity of total cofilin staining was apparently weaker than in tumors and immunoreactivity was negative in the basal cell layers of some (3/13) samples (Figure 2A). Staining for total cofilin predominated in the cytoplasm and for p-cofilin in the nuclei of normal cells (Figure 2A, B). No correlation between the percentage of cofilin or p-cofilin stained cells and clinicopathologic factors was observed.

**Figure 3:**
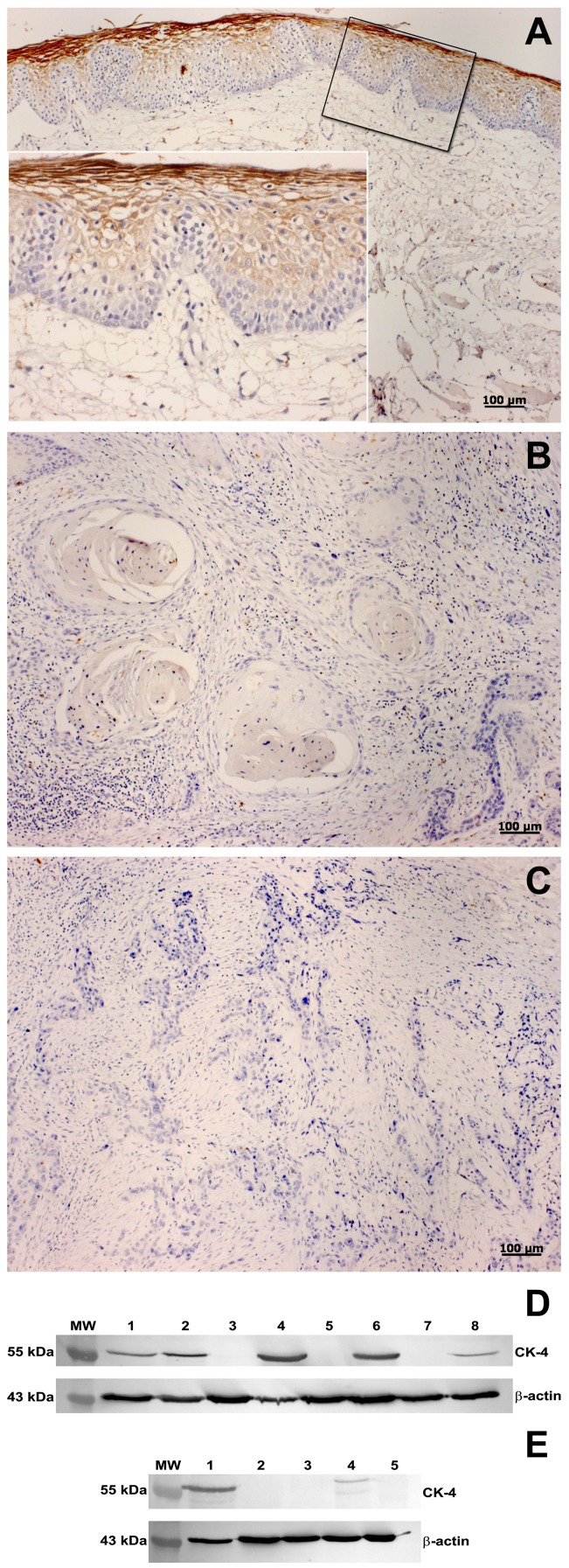
Immunodetection of keratin 4 expression in OSCC samples. Immunohistochemistry analysis: pattern of keratin 4 immunostaining in (A) superficial layers of epithelium in margin showing intense positivity in stratum corneum (A, insert); (B) absence of keratin 4 immunostaining in nests of well differentiated and (C) poorly differentiated areas of OSCC. Scale bar indicates 100 µm. **Western blot**: (D) tumor samples (lanes 1, 3, 5, 7) and matched margins (lanes 2, 4, 6, 8) from patients with T1N0, T4N2, T4N1 and T4N1 carcinomas, respectively; (E) Surgical margin (lane 1) and tumor samples (lanes 2, 3, 4, 5) from patients with T4N2, T4N2, T4N2, T1N0 and T2N2, respectively. β-actin was used as an internal control. MW, PageRuler™ Prestained Protein Ladder.

Confirming what was observed in 1-DE and 2-DE, keratin 4 immunodetection experiments showed consistent differences in expression in tumor samples compared with the surgical margins. In Western blotting, keratin 4 was negative (18/24) or showed low levels (3/24) in oral carcinomas and positive in most surgical margins (23/25) (Figure 3D–E).

The immunohistochemical analysis revealed keratin 4 expression in superficial layers of non-tumoral epithelium (margin) with intense positivity in stratum corneum (Figure 3A). Of the 65 surgical margins analyzed, 11 were keratin 4 negative independently of TNM: four with normal epithelium (4/33), two with hyperplasia (2/27) and five with dysplasia (5/5). Both patients with hyperplastic mucosa had a local recurrence 3 months or 3 years after surgery. Three cases showing dysplasia had a tumor relapse at the original site 3 to 10 months after surgery, and another developed a distant metastasis. Interestingly, among the patients exhibiting keratin 4-negative margins, two had a local recurrence 2 to 3 years after surgery and radiotherapy and also a distant metastasis; the other two patients developed a second primary tumor at the same anatomical site or a distant metastasis. Briefly, the frequency of recurrence, metastasis or a second primary tumor at the same site was 90.9% (10/11) in patients with keratin 4-negative margins in contrast to 51.8% (28/54) of the patients with keratin 4-positive margins (Fisher’s exact test; p = 0.0197).

Complete absence of keratin 4 immunostaining was detected in 72/72 tumors, even when well differentiated or poorly differentiated areas of OSCC were analyzed (Figure 3B–C).

### Knockdown of Cofilin-1 Inhibits Cell Invasion

**Figure 4:**
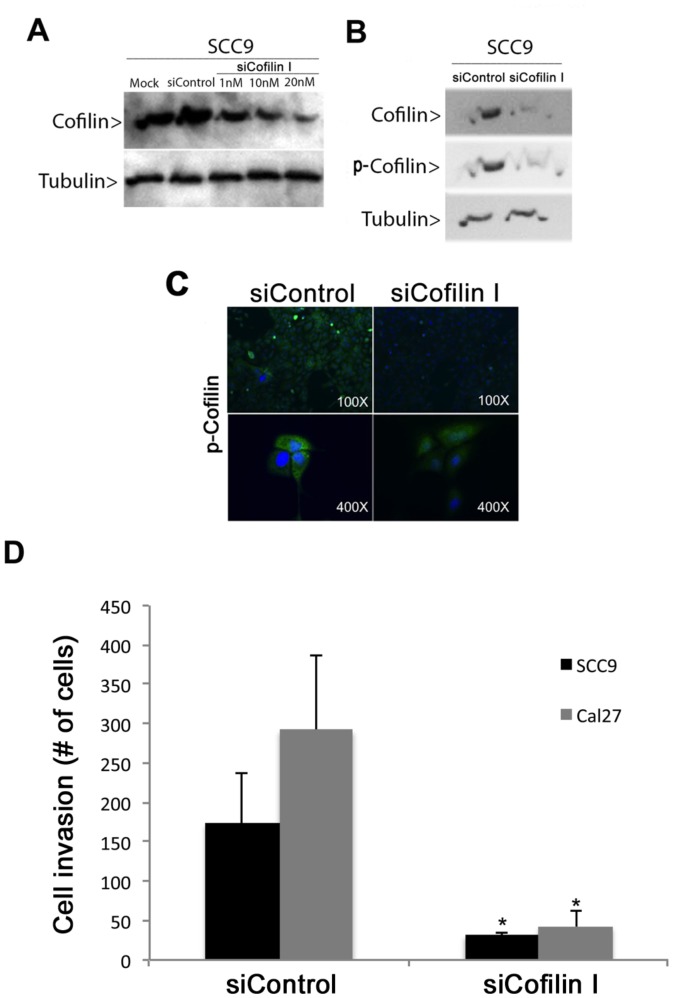
siRNA-mediated knockdown of cofilin-1 resulted in decreased invasive ability of oral cancer cells. Western blot analysis showed reduced levels of (A) cofilin-1 in ***SCC-9 cells*** *t*ransfected with different concentrations of siRNA (siCofilin I) for 48 h and of (B) cofilin-1 and p-cofilin in SCC-9 cells transfected with 20 nM siCofilin I for 48 h. (C) Immunofluorescence analysis of cofilin-1 knockdown SCC-9 cells (siCofilin I) using anti-p-cofilin antibody (green). (D) Invasion assays using Matrigel-coated filters were performed on SCC-9 and Cal 27 cells (cofilin-1 knockdown cells and controls). Bar graph represents the mean ± S.E. of the number cells that invaded through the Matrigel from three independent experiments (Student’s *t* test, * = p<0.01).

To clarify the role of cofilin-1 on oral cancer cell invasion, the expression of this protein was knocked down using siRNA. Three siRNAs with sequence complementarity to cofilin-1 transcripts (siCofilin I, II and III) were tested in SCC-9 cells and apparently exhibited similar functional potency. As shown in Figure 4A–C, both Western blot and immunofluorescence analyses confirmed down-regulation of total and p-cofilin protein expression in specific RNAi-treated cells, but not in control cells.

In the Boyden chamber assay, which mimics invasion of cancer cells across a basement membrane, the number of cofilin-knocked down SCC-9 and CAL 27 cells that invaded across the Matrigel membrane was decreased compared to cells transfected with control siRNA (SCC-9: mean number of cells/field  =  31.5 versus 173.5, Student’s *t* test, p<0.001; CAL 27: mean number of cells/field  =  42.3 versus 292, Student’s *t* test, p<0.05) (Figure 4D). The results suggest that cofilin-1 overexpression may be important for oral tumor cell invasion during the metastatic process.

## Discussion

Oral squamous cell carcinoma is a heterogeneous disease, both at the histopathological and molecular level, and in its response to therapy. Accurate assignment of patients to specific subgroups is apparently critical to predicting outcome and hence to guide therapeutic decisions and improve survival in OSCC and other head and neck carcinomas. An initial genetic classification model has recently been proposed for this group of diseases by Leemans et al. [1] using a limited number of parameters to separate classes with different prognosis. Actually, many clinicopathological variables and genetic/epigenetic events related to head and neck tumorigenesis have been described and associated with reduced survival or more-aggressive tumor behavior (reviewed by [29] and [1]). They include traditional histopathological criteria, such as the presence of lymph node metastases, which unfortunately is limited in predicting outcome. A plethora of potential molecular markers in OSCC have also been reported, although with a wide heterogeneity in prognostic significance. The more frequently cited are inactivation of tumor suppressor p53, p16^INK4a^ and PTEN (phosphatase and tensin homolog) proteins, and activation or overexpression of cyclin D, EGFR (epidermal growth factor receptor), MET (hepatocyte growth factor receptor) and VEGF (vascular endothelial growth factor) [1].

In the present study, we used exploratory parameters for classifying oral carcinomas with regard to protein expression profile, and analyzed a large set of OSCC samples by 1-DE, 2-DE, MS and immunodetection methods in order to contribute to the molecular characterization of OSCC subgroups with risk of tumor dissemination. Differential expression was evaluated using small but lymph node metastasis-positive and large, lymph node metastasis-negative tumors, and surgical margins. The patient group was homogeneous in relation to age and tobacco and alcohol use, therefore, potentially presenting similar etiologic factors.

Although divergent in number of proteins and strategies to obtain the relative quantification values, 1-DE and 2-DE expression patterns were consistent with each other, particularly in the comparison between tumors and their surgical margins. Some dissimilarity was observed between 1-DE and 2-DE expression patterns from “more-aggressive” and “less-aggressive” tumors, likely reflecting differences between the compositions of pools: whereas the LA group analyzed by 1-DE included only T3N0 cases, LA group analyzed by 2-DE also included T2N0.

In general, the parameters defined by the present study for classifying oral carcinomas revealed groups with distinctive protein expression patterns that may be related to different tumor behaviors. A number of proteins identified have already been observed in OSCCs, but many others were not yet described as deregulated in these tumors, at least to our knowledge, *inter alia* asporin, ANP32A protein, α-actinin-1, four and a half LIM domains protein 1, myozenin-1, nascent polypeptide-associated complex subunit α, mimecan, serine/arginine-rich splicing factor 3, voltage-dependent anion-selective channel protein 1 and 14-3-3 protein β/α. Some of them are related to actin cytoskeleton, cellular movement and cell growth or signaling. Also important was the observation that the genes coding for several proteins overexpressed in tumors (both MA and LA) have been mapped to 11q13 (*CFL1*, *GSTP1*, *SERPINH1*) and 8q23-24 (*EEF1D*, *RPL8*), which are chromosome segments frequently amplified in head and neck carcinomas and may be related to the progression of the disease [30], [31]. Only cofilin-1 [32]–[34] and glutathione S-transferase P (or GSTP1-1) [33], [35] have been previously described in OSCC; otherwise, few studies have been done on *SERPINH1*, *EEF1D* and *RPL8* expression in head and neck carcinomas or other neoplasms.

Concerning differences between tumors and margins, many proteins were identified, some previously associated with OSCC, validating our approach. As expected, those related to signaling cascades frequently altered in cancer cells were deregulated in our tumor samples, such as stratifin (p53 pathway), heat shock protein β1, 14-3-3 protein β/α, Ras-related protein Rap-1A, stathmin (EGFR, Ras and MAPKK pathway), asporin (TGFβ pathway), 40S ribosomal protein S6 (mTOR pathway), heat shock protein β1 (VEGF pathway), collagen α-1 I (WNT pathway).

Our results also included several proteins involved in keratinocyte differentiation and epidermis development. We noted a keratin profile compatible with loss of differentiation in tumors: increased expression of proteins typical of stem cells or transient amplifying cells and loss of a keratin characteristic of differentiating layers of epithelia (keratin 4) [36]. These findings shed light on the importance of the keratinization process in oral cancer and of identifying differentially expressed keratins or related proteins, which may be useful for the development of new diagnostic tests and treatments.

In 1-DE and 2-DE experiments, the absence or underexpression of keratin 4 in tumor samples was a consistent finding, confirming the results of several authors [37]–[39] and validating the strategies used in the present study. Similar results were obtained by Western blot in most tumors. The complete absence of keratin 4 was also observed in all tumors analyzed by immunohistochemistry and in a few surgical margins. Patients exhibiting keratin 4-negative margins showed higher rates of local recurrence, metastasis or a second primary tumor at the same anatomical site than patients with keratin 4-positive margins. Interestingly, all five dysplastic margins, which may represent a tumor precursor field or the presence of residual cancer cells, were keratin 4 negative; four of the patients developed tumor relapse or a distant metastasis. These findings are in accordance with the data of Schaaij-Visser et al. [39] and support the recommendation that low immunoexpression of keratin-4 in surgical margins may qualify patients at high risk of tumor recurrence for more stringent surveillance protocols.

With respect to tumor behavior, specific protein patterns favoring metastasis were related to the “more-aggressive” group defined by the present study. This group displayed upregulation of proteins involved in migration, adhesion, angiogenesis, cell cycle regulation, anti-apoptosis and epithelial to mesenchymal transition, which are consistent with a metastatic phenotype, whereas cells of the “less-aggressive” group were engaged in keratinocyte differentiation, epidermis development, inflammation and immune response.

A remarkable finding of the present study was the involvement of cofilin pathway in the aggressive phenotype, which requires reorganization of the cytoskeleton and migratory features. Cofilin-1, a small protein able to bind actin, has been previously shown to be overexpressed in some tumors [40]–[42]. In oral carcinomas, few studies have observed altered expression of cofilin [32]–[34] but no reference about its role on oral cancer cell invasion has been published. This protein, its isoforms and regulators play a central role in the actin filament remodeling and are important factors directly involved in chemotaxis, cell migration and metastasis [43].

**Figure 5:**
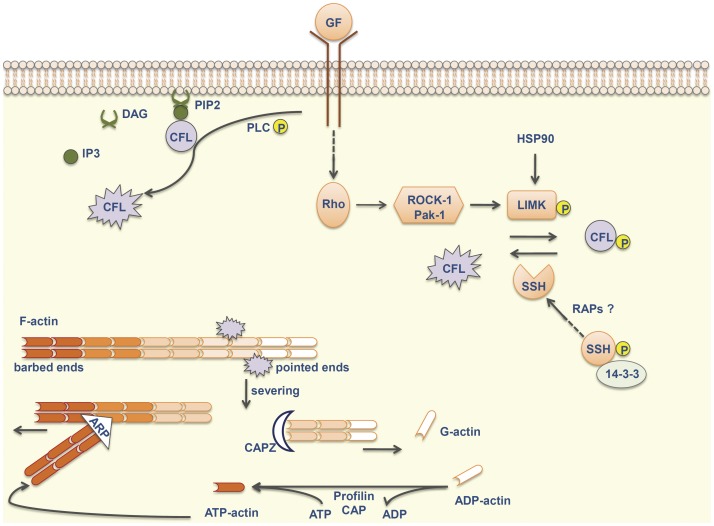
Cofilin pathway. Microenvironmental stimuli signal through Rho-GTPases and their regulating kinases (ROCK1 and Pak-1), stimulating LIMK to phosphorylate and inactivate cofilin-1. Otherwise, SSH phosphatases dephosphorylate cofilin. Rap proteins may increase the enzymatic activity of SSHs, possibly by promoting their release from 14-3-3 proteins. Cofilin is sequestered by PIP2 and released after hydrolysis of PIP2 by phosphorylated PLC to IP3 and DAG. The active cofilin severs “old” actin filaments to generate free actin barbed ends. ATP-actin assembles into these barbed ends and ADP-actin subunits are, in turn, dissociated from the pointed end. Free actin monomers exchange ADP to ATP, frequently with the help of profilin and CAP proteins. ARP2/3 complex binds to F-actin and nucleates the growth of daughter filaments, generating a dendritic network at the leading edge of migratory cells. Other members of this pathway include Hsp90, which promotes stability of LIMK, and CAPZ, which interacts with barbed ends and inhibits filament assembly. ARP = actin-related protein 2/3 complex; CAP = adenylyl cyclase-associated protein 1; CAPZ = F-actin-capping protein subunit alpha-1; CFL = cofilin-1; F-actin = filamentous actin; DAG =  diacylglycerol; G-actin = globular actin; GF = growth factor; HSP90 = heat shock protein HSP 90-alpha; IP3 = inositoltrisphosphate; LIMK = LIM kinases; Pak-1 = serine/threonine-protein kinase PAK 1; PIP2 = phosphatidylinositol-4-5-biphosphate; PLC = phospholipase C; RAP = Ras-related protein; ROCK-1 = Rho-associated protein kinase 1; SSH = slingshot phosphatase.

The cofilin pathway is activated by growth factors that signal through Rho-GTPases and several kinases, such as ROCK-1 and Pak-1, stimulating LIM kinases (LIMKs) to phosphorylate (and thereby inactivate) cofilin-1 on Ser3. Cofilin-1 activity is also modulated by increasing pH, by dephosphorylation through phosphatases (slingshot or SSHs and chronophin) and by PLCγ1-dependent hydrolysis of phosphatidylinositol-4-5-biphosphate (PIP2) to which it is bound at the plasma membrane (Figure 5). Activated cofilin-1 severs “old” actin filaments (F-actin) to generate globular actin (G-actin) and free actin barbed ends. ATP-G-actin assembles into the barbed end and ATP is hydrolyzed generating ADP-actin subunits, which are, in turn, dissociated from the pointed end of “old” filaments, in a process called treadmilling. Free G-actin monomers exchange ADP to ATP, frequently with the help of profilin, and are again incorporated into filaments (reviewed by [43], [44] and [31]).

Cofilin competes with actin-related protein ARP2/3 [45], a protein complex that binds to the side of F-actin and nucleates the growth of daughter filaments at a 70° angle to the mother filament, generating a dendritic network at the leading edge of migratory cells [46]. The dynamic assembly at barbed ends/disassembly at pointed ends of actin filaments and branching are required to push the cellular membrane forward resulting in protrusion and migration.

There are many other scaffolding activators or partners indirectly involved in the cofilin pathway [47]. For example, 14-3-3 proteins inhibit SHH [48], whereas RAP proteins increase the enzymatic activity of these phosphatases through different effectors [49], upregulating non-phosphorylated cofilin levels. Otherwise, Hsp90 promotes stability of LIMK, which may result in increased levels of phospho-cofilin [50]. Capping proteins interact with barbed ends and inhibit filament assembly [51] whereas CAP1 mediates actin filament turnover [52]. Actin dynamics may also be regulated by myosins [53], septins [54], and by tropomyosins in an isoform-specific way [55].

In the present study, most members of the cofilin pathway exhibited over (cofilin-1, CAP1, F-actin-capping protein α-1, HSP 90-α, tropomyosin 3) or underexpression (ARP2/3 subunit 2) in “more-aggressive” tumors analyzed by 1-DE, 2-DE and MS/MS, as well in tumors compared to surgical margins (ARP2/3 subunit 2, cofilin-1, CAP1, HSP 90-α, F-actin-capping protein α-1 or CAPZA1, Rap-1A, 14-3-3 protein β/α, profilin-1, tropomyosins). By immunohistochemical analysis, total cofilin-1 and p-cofilin apparently also showed higher expression in tumor than in normal epithelium and, although the differences were not large, the results agreed with those obtained by our proteomic experiments and by other authors [32]–[34]. Unlike what was observed by 2-DE, differences in immunoreactivity of cofilin were not evident between MA and LA cases and the data call for validation in a larger set of cases.

*Altered* cofilin/actin ratio and even slight variations in cofilin levels may affect cell mobility. However, it is hard to have a conclusion on the role of cofilin and its partners on the metastatic behavior only from semi-quantitative analyses of their expression, *without taking into account the differences* over time or between specific subcellular compartments *(*reviewed by [56] *).*

Our results showed total cofilin-1 predominantly localized in the cytoplasm and p-cofilin in the cytoplasm and nuclei of tumor cells. The model by Chhabra and dos Remedios [57] explains the presence of nuclear p–cofilin and suggests its indirect role in gene expression. These authors proposed that cofilin transports ADP-actin subunits into the nucleus, where the higher ATP concentration promotes the exchange of ADP for ATP. After disruption of the cofilin-actin complex, probably by nuclear LIMK-dependent phosphorylation of cofilin, ATP-actin monomers can self-assemble into oligomers and influence chromatin remodeling or gene expression (reviewed by [58]).

Cofilin-1 knockdown by siRNA significantly inhibited oral cancer cell invasion across Matrigel *in vitro*. This finding agrees with the fact that cofilin-1 is involved in the formation of invadopodia [59], which are actin-rich membrane protrusions of invasive cells with extracellular matrix degradation activity [53], [60]. Downregulation of cofilin expression inhibits the stability of invadopodia [59], pointing out its role in cell invasion.

Our results indicate that the cofilin pathway is deregulated in oral carcinomas and related to aggressive behavior. Taking into account the definition proposed by Leemans et al. [1], *CFL1* may be considered an established cancer gene since (a) it is mapped at 11q13, a chromosome region that is amplified in head and neck tumors and related to invasion, metastasis and decreased disease free survival [31], [61]; (b) knock down of *CFL1* expression inhibits oral cancer cell invasion *in vitro* (a cancer-associated phenotype), which hints at an oncogenic function; and finally, (c) CFL1 protein is activated by EGFR and its effectors [43], a signaling pathway with several established cancer genes related to oral cancer, such as *EGFR* itself, *PTEN* and *CCND1* [1].

### Conclusion

To our knowledge, this is the first proteomic study of oral carcinomas that compares small but lymph node metastasis-positive and large, lymph node metastasis-negative tumors. We observed that these criteria separate distinctive protein expression patterns and that the tumor size parameter might improve the detection of proteins associated with an aggressive behavior.

Besides the identification of several proteins not yet described as deregulated in oral cancer, the present study demonstrated for the first time the role of cofilin-1 in modulating OSCC cell invasion, adding new data that may be useful for predicting aggressive phenotype in OSCC.

## Supporting Information

Figure S1 **Partial 2-DE gel images of proteins from** ***“more-aggressive” (T1-2N+)*** **OSCC tumors and surgical margins.** Myosin light chain 1/3, skeletal muscle isoform (MLC1/MLC3); beta-globin (HBB); carbonic anhydrase 3 (CA-III); creatine kinase M-type (M-CK); keratin 4 (CK-4); gamma-actin (ACTG); myoglobin (MB); myosin light chain 3 (MYL3); myosin regulatory light chain 2, skeletal muscle isoform (MLC2B); myosin regulatory light chain 2, ventricular/cardiac muscle isoform (MLC-2v); tropomyosin-1 (TPM1); tropomyosin-2 (TPM2); tropomyosin-3 (TPM3); alpha-enolase (ENO1); cofilin-1 (CFL1); cyclophilin A (PPIase A); keratin 19 (CK-19); glutathione S-transferase P (GSTP1-1); heat shock 27 kDa (HSP 27); calgranulin-B (S100-A9); serum albumin (ALB); stratifin (SFN); superoxide dismutase [Cu-Zn] (SOD1); tropomyosin-4 (TPM4); vimentin (VIM). Tumors and matched surgical margins from tongue (C02) and floor of mouth (C04).
(TIF)

Figure S2 **Partial 2-DE gel images of proteins from** ***“less-aggressive”*** ** ***(*** **T2-3N0) OSCC tumors and surgical margins.** Myosin light chain 1/3, skeletal muscle isoform (MLC1/MLC3); annexin A1 (ANXA1); carbonic anhydrase 3 (CA-III); creatine kinase M-type (M-CK); myoglobin (MB); myosin light chain 3 (MYL3); myosin regulatory light chain 2, skeletal muscle isoform (MLC2B); myosin regulatory light chain 2, ventricular/cardiac muscle isoform (MLC-2v); tropomyosin-1 (TPM1); tropomyosin-2 (TPM2); tropomyosin-3 (TPM3); troponin T, slow skeletal muscle (TnTs); alpha-enolase (ENO1); cofilin-1 (CFL1); cyclophilin A (PPIase A); keratin 19 (CK-19); galectin-7 (Gal-7); heat shock 27 kDa (HSP 27); stratifin (SFN); tropomyosin-4 (TPM4). Tumors and matched surgical margins from tongue (C02) and floor of mouth (C04).
(TIF)

Figure S3 **Partial 2-DE gel images of proteins from** ***“more-aggressive” (T1-2N+) and “less-aggressive”*** ** ***(*** **T2-3N0)** **OSCC tumors.** Alpha-enolase (ENO1); annexin A2 (ANXA2); cofilin-1 (CFL1); glutathione S-transferase P (GSTP1-1); keratin 19 (CK-19); myoglobin (MB); stratifin (SFN); superoxide dismutase (SOD1). Tumor samples from tongue (C02) and floor of mouth (C04).
(TIF)

Table S1 **Clinicopathological features of 144 patients with OSCC and techniques used to analyze the samples.** T = tumor; Ma = surgical margin; F = female; M = male; P or Neg = positive or negative exposition to tobacco/alcohol, respectively, but consumption time is indeterminate; 1-DE = one-dimensional gel electrophoresis; 2-DE = two-dimensional gel electrophoresis; WB = Western blot; IH = immunohistochemistry; NA = not available.
(DOC)

Table S2 **Pools organized into** ***groups according*** **to TNM system.** 1-DE = one-dimensional gel electrophoresis; 2-DE = two-dimensional gel electrophoresis.
(DOC)

Table S3 **Protein identification in “more-aggressive” (MA), “less-aggressive” (LA) tumor groups and their surgical margins (SM) by 1-DE and Scaffold software according to quantitative value.** Proteins with a fold change of at least 1.5 were considered with differential abundance between the groups.
(PDF)

Table S4 **Differentially expressed proteins identified by 2-DE followed by mass spectrometry analysis in “more-aggressive” (MA), “less-aggressive” (LA) tumor groups and their surgical margins (SM).** MA/SM, LA/SM and MA/LA abundance ratio.
(DOC)
