# Supplementary figures and images for: Proteomic Approaches Identify Members of Cofilin Pathway Involved in Oral Tumorigenesis
Source: PLoS One. 2012 Dec 5;7(12):e50517. doi: 10.1371/journal.pone.0050517 (PMC3515627; doi:10.1371/journal.pone.0050517)

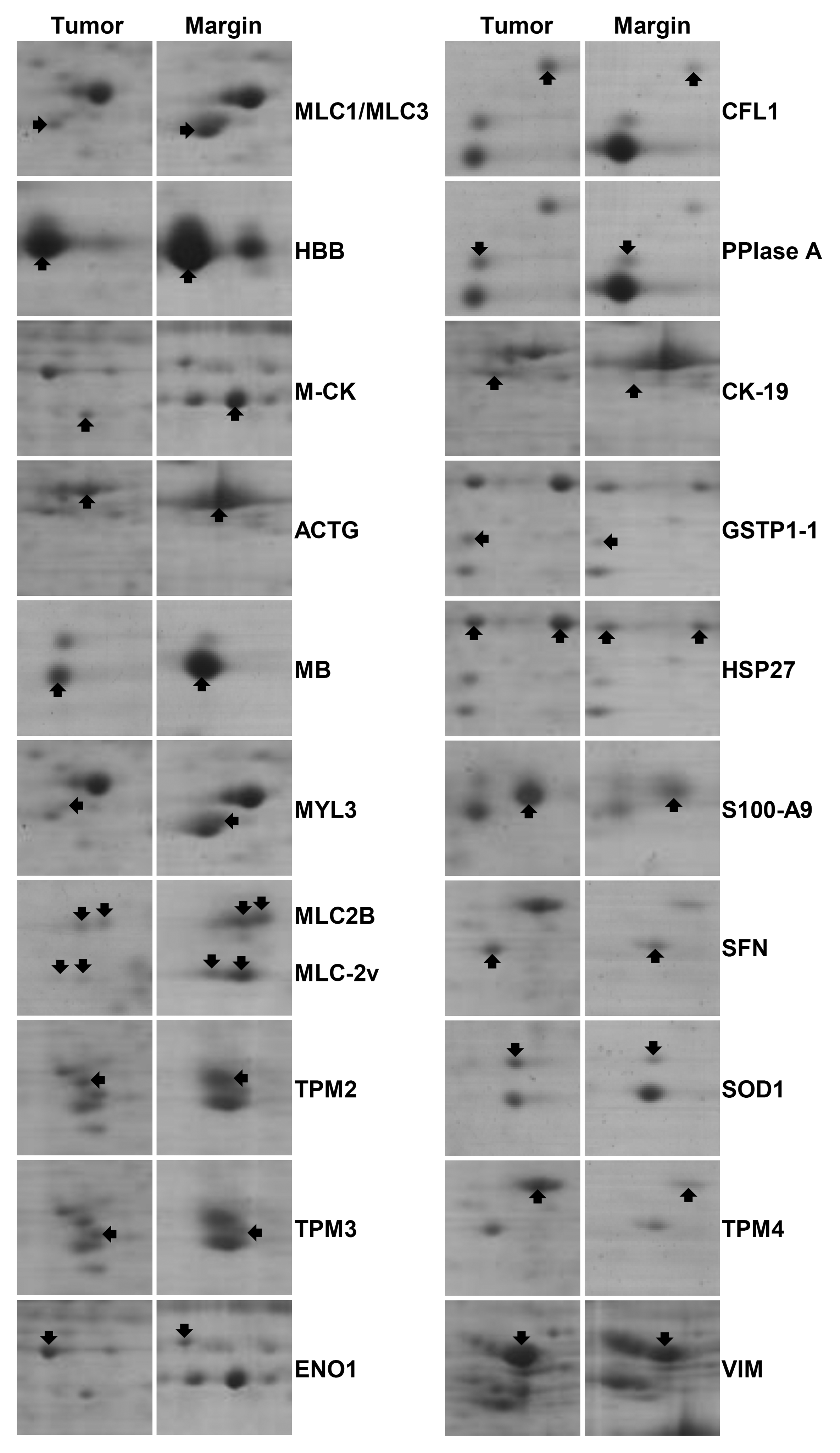

Supplement: Figure S1 — Partial 2-DE gel images of proteins from “more-aggressive” (T1-2N+) OSCC tumors and surgical margins. Myosin light chain 1/3, skeletal muscle isoform (MLC1/MLC3); beta-globin (HBB); carbonic anhydrase 3 (CA-III); creatine kinase M-type (M-CK); keratin 4 (CK-4); gamma-actin (ACTG); myoglobin (MB); myosin light chain 3 (MYL3); myosin regulatory light chain 2, skeletal muscle isoform (MLC2B); myosin regulatory light chain 2, ventricular/cardiac muscle isoform (MLC-2v); tropomyosin-1 (TPM1); tropomyosin-2 (TPM2); tropomyosin-3 (TPM3); alpha-enolase (ENO1); cofilin-1 (CFL1); cyclophilin A (PPIase A); keratin 19 (CK-19); glutathione S-transferase P (GSTP1-1); heat shock 27 kDa (HSP 27); calgranulin-B (S100-A9); serum albumin (ALB); stratifin (SFN); superoxide dismutase [Cu-Zn] (SOD1); tropomyosin-4 (TPM4); vimentin (VIM). Tumors and matched surgical margins from tongue (C02) and floor of mouth (C04). (TIF) [file pone.0050517.s001.tif]

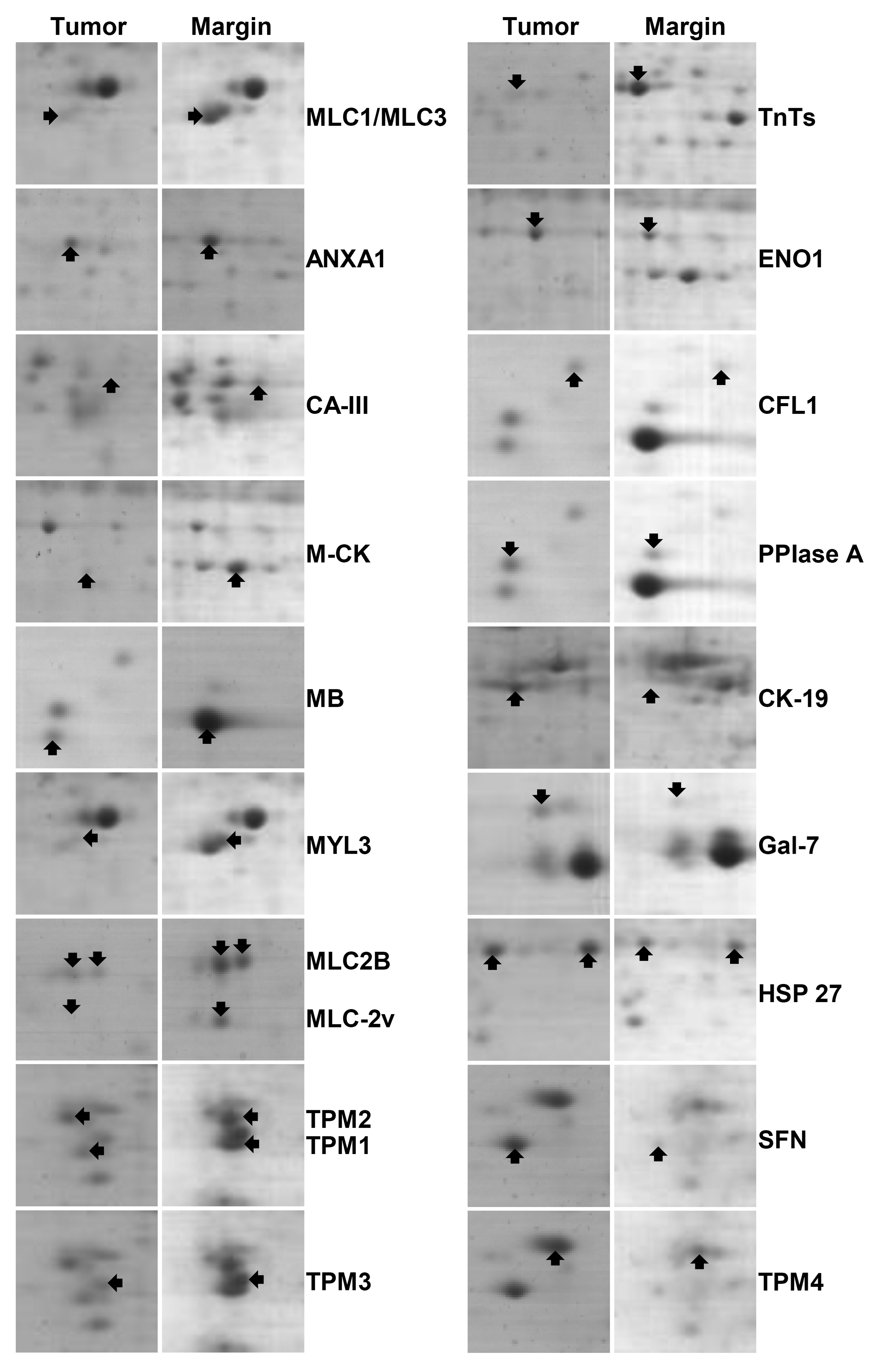

Supplement: Figure S2 — Partial 2-DE gel images of proteins from “less-aggressive” ( T2-3N0) OSCC tumors and surgical margins. Myosin light chain 1/3, skeletal muscle isoform (MLC1/MLC3); annexin A1 (ANXA1); carbonic anhydrase 3 (CA-III); creatine kinase M-type (M-CK); myoglobin (MB); myosin light chain 3 (MYL3); myosin regulatory light chain 2, skeletal muscle isoform (MLC2B); myosin regulatory light chain 2, ventricular/cardiac muscle isoform (MLC-2v); tropomyosin-1 (TPM1); tropomyosin-2 (TPM2); tropomyosin-3 (TPM3); troponin T, slow skeletal muscle (TnTs); alpha-enolase (ENO1); cofilin-1 (CFL1); cyclophilin A (PPIase A); keratin 19 (CK-19); galectin-7 (Gal-7); heat shock 27 kDa (HSP 27); stratifin (SFN); tropomyosin-4 (TPM4). Tumors and matched surgical margins from tongue (C02) and floor of mouth (C04). (TIF) [file pone.0050517.s002.tif]

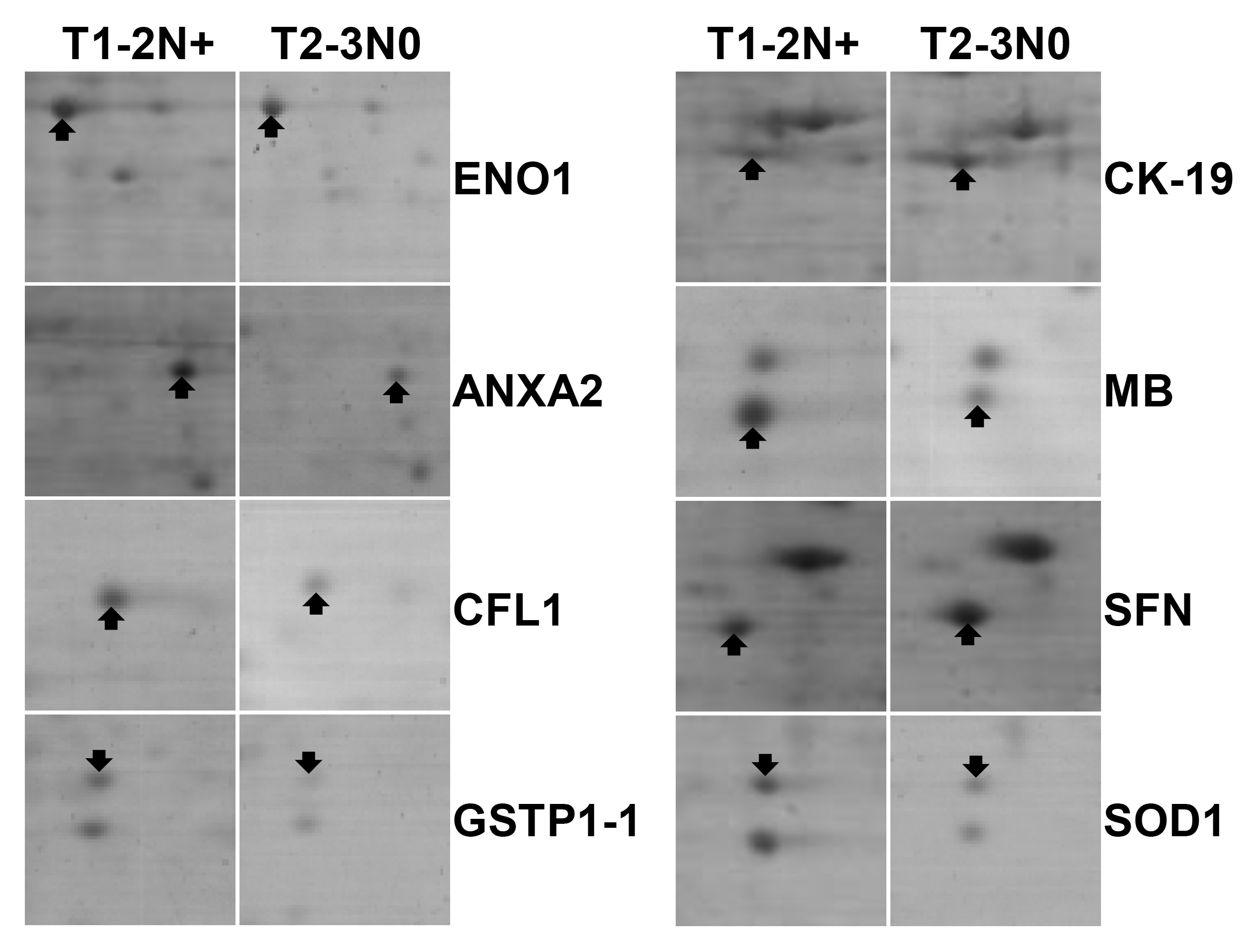

Supplement: Figure S3 — Partial 2-DE gel images of proteins from “more-aggressive” (T1-2N+) and “less-aggressive” ( T2-3N0) OSCC tumors. Alpha-enolase (ENO1); annexin A2 (ANXA2); cofilin-1 (CFL1); glutathione S-transferase P (GSTP1-1); keratin 19 (CK-19); myoglobin (MB); stratifin (SFN); superoxide dismutase (SOD1). Tumor samples from tongue (C02) and floor of mouth (C04). (TIF) [file pone.0050517.s003.tif]
