# Supplementary material for: Proteomic Approaches Identify Members of Cofilin Pathway Involved in Oral Tumorigenesis
Source: PLoS One. 2012 Dec 5;7(12):e50517. doi: 10.1371/journal.pone.0050517 (PMC3515627; doi:10.1371/journal.pone.0050517)
Supplement: Table S1 — Clinicopathological features of 144 patients with OSCC and techniques used to analyze the samples. T = tumor; Ma = surgical margin; F = female; M = male; P or Neg = positive or negative exposition to tobacco/alcohol, respectively, but consumption time is indeterminate; 1-DE = one-dimensional gel electrophoresis; 2-DE = two-dimensional gel electrophoresis; WB = Western blot; IH = immunohistochemistry; NA = not available. (DOC) [file pone.0050517.s004.doc]

**Supporting Table 1. Clinicopathological features of 144 patients with OSCC and techniques used to analyze the samples.** T=tumor; Ma=surgical margin; F=female; M=male; P or Neg=positive or negative exposition to tobacco or alcohol, respectively, but consumption time is indeterminate; 1-DE=one-dimensional electrophoresis; 2-DE=two-dimensional electrophoresis; WB=Western blot; IH=immunohistochemistry; NA=not available.

| **Case** | **Type of sample** | **Site^a^** | **Gender / Age (yrs)** | **Pathologic stage** | **Histological differentiation** | **Lifetime smoking (yrs)** | **Lifetime alcohol consumption (yrs)** | **Technique** |
| --- | --- | --- | --- | --- | --- | --- | --- | --- |
| CP1/0151 | T/Ma | C02 | M/47 | T3N0M0 | Moderate | 33 | 33 | 1-DE |
| CP1/0191 | T | C04 | M/63 | T3N0M0 | Moderate | 47 | 47 | 1-DE |
| CP1/0262 | Ma | C02 | M/62 | T3N0M0 | Moderate | 34 | 44 | 1-DE |
| CP2/0051 | T/Ma | C04 | M/46 | T2N2bM0 | Well | 33 | 10 | 1-DE |
| CP3/0280 | T/Ma | C04 | M/52 | T1N2b | Moderate | 38 | 27 | 1-DE |
| CP3/0292 | T/Ma | C04 | M/50 | T2N2c | Moderate | 34 | 25 | 1-DE |
| CP3/0139 | T | C02 | M/50 | T2N2aM0 | Moderate | 37 | 37 | 1-DE/2-DE |
| CP2/0132 | T/Ma | C02 | M/48 | T2N1M0 | Moderate | 33 | 31 | 1-DE/IH |
| CP1/0031 | T/Ma | C04 | M/50 | T3N0M0 | Well | 30 | 30 | 1-DE/WB |
| CP1/0277 | T/Ma | C02 | M/66 | T3N0M0 | Moderate | 45 | 35 | 1-DE/WB |
| CP1/0283 | T/Ma | C04 | M/49 | T3N0M0 | Well | 21 | 15 | 1-DE/WB |
| CP1/0017 | T/Ma | C02 | M/55 | T2N0M0 | Moderate | 45 | 29 | 2-DE |
| CP1/0051 | Ma | C04 | M/62 | T2N0M0 | Moderate | 47 | 44 | 2-DE |
| CP1/0075 | T/Ma | C04 | M/54 | T3N0M0 | Moderate | 33 | 33 | 2-DE |
| CP1/0080 | T/Ma | C04 | M/67 | T3N0M0 | Well | 52 | 49 | 2-DE |
| CP1/0083 | T/Ma | C04 | M/72 | T2N0M0 | Well | 36 | 34 | 2-DE |
| CP1/0094 | T/Ma | C04 | M/44 | T2N2BM0 | Well | 27 | 27 | 2-DE |
| CP2/0175 | Ma | C04 | M/61 | T2N2bM0 | Moderate | 43 | 41 | 2-DE |
| CP2/1004 | Ma | C04 | F/44 | T2N0M0 | Well | 20 | 6 | 2-DE |
| CP3/0004 | T | C04 | M/60 | T2N0M0 | Well | 26 | 32 | 2-DE |
| CP3/0012 | T/Ma | C04 | M/50 | T1N1M0 | Well | 13 | 18 | 2-DE |
| CP3/0046 | T | C02 | F/75 | T2N2bM0 | Moderate | 23 | 67 | 2-DE |
| CP3/0049 | T/Ma | C02 | M/62 | T2N2bM0 | Moderate | 34 | 37 | 2-DE |
| CP3/0050 | T/Ma | C02 | F/44 | T2N1M0 | Well | 26 | 17 | 2-DE |
| CP3/0083 | T/Ma | C02 | F/55 | T1N1M0 | Moderate | 46 | 40 | 2-DE |
| CP3/0087 | T/Ma | C02 | M/52 | T2N2cM0 | Moderate | 40 | 37 | 2-DE |
| CP3/0101 | T/Ma | C04 | M/62 | T3N0M0 | Moderate | 52 | 30 | 2-DE |
| CP3/0120 | T/Ma | C04 | F/46 | T2N0M0 | Well | 31 | 31 | 2-DE |
| CP3/0138 | T | C04 | M/75 | T2N0M0 | Moderate | 53 | 45 | 2-DE |
| CP3/0193 | T | C02 | M/49 | T2N1M0 | Well | 10 | 15 | 2-DE |
| CP2/0093 | T/Ma | C04 | M/55 | T2N0M0 | Well | 37 | 37 | 2-DE/IH |
| CP2/0120 | T/Ma | C02 | F/41 | T2N1M0 | Moderate | 24 | Neg | 2-DE/IH |
| CP2/0185 | T/Ma | C02 | M/64 | T2N0M0 | Moderate | 35 | 45 | 2-DE/IH |
| 259504 | T | C04 | F/61 | T4N3M0 | NA | NA | NA | IH |
| 259708 | T | C02 | M/54 | T4N2cM0 | NA | P | P | IH |
| 267108 | T | C04 | M/50 | T1N0M0 | NA | P | P | IH |
| 267343 | T/Ma | C02 | M/38 | T3N2BM0 | NA | P | P | IH |
| 267553 | T/Ma | C02 | M/62 | T4N2AM0 | NA | P | P | IH |
| 267931 | T | C02 | M/49 | T4N2BM0 | NA | P | P | IH |
| 268616 | T | C02 | F/57 | T2N1M0c | NA | P | P | IH |
| 268783 | T | C04 | M/52 | T2N0M0 | NA | P | P | IH |
| 268786 | T | C02 | M/70 | T4N0M0 | NA | P | P | IH |
| 269330 | T/Ma | C04 | M/48 | T3N2BM0 | NA | P | P | IH |
| 270293 | T/Ma | C03 | M/73 | T4N0M0 | NA | P | P | IH |
| 270340 | T | C04 | M/62 | T4N0M0 | NA | P | P | IH |
| 281294 | T/Ma | C02 | M/60 | T2N0M0 | NA | P | Neg | IH |
| 287806 | Ma | C04 | M/46 | T2N2bM0 | NA | P | P | IH |
| 295618 | T/Ma | C04 | M/NA | T2N3M0 | NA | NA | NA | IH |
| 297562 | T/Ma | C04 | M/68 | T4N3M0 | NA | P | P | IH |
| 298686 | T/Ma | C04 | M/55 | T2N0M0 | NA | P | P | IH |
| CP2/0003 | T/Ma | C03 | F/79 | T2N1M0 | Moderate | 72 | Neg | IH |
| CP2/0008 | T/Ma | C02 | M/54 | T2N0M0 | Moderate | 26 | 24 | IH |
| CP2/0010 | T/Ma | C06 | M/55 | T3N0Mx | Well | 38 | P | IH |
| CP2/0013 | T/Ma | C02 | M/68 | T4N2cM0 | Moderate | 50 | 50 | IH |
| CP2/0019 | T/Ma | C04 | M/63 | T3N2M0 | Moderate | 45 | 43 | IH |
| CP2/0023 | T/Ma | C02 | M/54 | T3N0M0 | Moderate | 41 | 34 | IH |
| CP2/0029 | T | C04 | M/70 | T2N0M0 | Moderate | 56 | 54 | IH |
| CP2/0036 | T/Ma | C06 | M/55 | T4N2b M0 | Well | 39 | 39 | IH |
| CP2/0039 | T | C02 | M/56 | T2N0M0 | Well | 38 | 35 | IH |
| CP2/0040 | T/Ma | C02 | M/41 | T1N2bM0 | Poor | Neg | Neg | IH |
| CP2/0071 | T/Ma | C04 | M/49 | T4N2bM0 | Well | 36 | 34 | IH |
| CP2/0074 | T/Ma | C04 | M/40 | T4N0M0 | Well | 32 | 30 | IH |
| CP2/0081 | T | C03 | F/62 | T4N0M0 | Poor | 26 | Neg | IH |
| CP2/0087 | T/Ma | C03 | M/69 | T4N2bM0 | Poor | 27 | 52 | IH |
| CP2/0094 | T/Ma | C02 | M/47 | T3N1M0 | Poor | 30 | 30 | IH |
| CP2/0109 | T/Ma | C04 | M/63 | T3N0M0 | Well | 15 | 45 | IH |
| CP2/0114 | T/Ma | C04 | M/51 | T4N2M0 | Moderate | 22 | 41 | IH |
| CP2/0115 | T/Ma | C06 | M/63 | T4N0M0 | Well | 43 | 28 | IH |
| CP2/0116 | T/Ma | C04 | F/81 | T2N0M0 | Well | 5 | Neg | IH |
| CP2/0117 | T/Ma | C02 | M/35 | T2N2bM0 | Moderate | 28 | 17 | IH |
| CP2/0118 | T/Ma | C02 | M/45 | T2N1M0 | Well | 23 | 12 | IH |
| CP2/0122 | T/Ma | C04 | M/56 | T2N2bM0 | Well | 40 | 40 | IH |
| CP2/0125 | T/Ma | C03 | M/53 | T4N0M0 | Well | 41 | 41 | IH |
| CP2/0130 | T/Ma | C03 | M/49 | T2N0M0 | NA | 31 | 29 | IH |
| CP2/0133 | T/Ma | C02 | M/46 | T4N1M0 | Moderate | 32 | 28 | IH |
| CP2/0144 | T/Ma | C06 | M/65 | T2N0M0 | Moderate | 43 | 43 | IH |
| CP2/0149 | T/Ma | C04 | M/58 | T4N1M0 | Moderate | 50 | 40 | IH |
| CP2/0152 | T/Ma | C02 | M/46 | T4N2bM0 | Moderate | 33 | 33 | IH |
| CP2/0166 | T/Ma | C03 | F/62 | T4N2bM0 | Well | 2 | Neg | IH |
| CP2/0168 | T/Ma | C04 | F/70 | T2N0M0 | Well | 4 | Neg | IH |
| CP2/0169 | T/Ma | C04 | M/42 | T2N2bM0 | Moderate | 32 | 14 | IH |
| CP2/0170 | T/Ma | C04 | M/34 | T4N2bM0 | Moderate | 20 | 20 | IH |
| CP2/0177 | T/Ma | C02 | M/45 | T3N0M0 | Well | Neg | 25 | IH |
| CP2/0181 | T/Ma | C04 | M/62 | T4N1M0 | Well | 55 | 45 | IH |
| CP2/0182 | T/Ma | C06 | M/38 | T4N0M0 | Moderate | 14 | 14 | IH |
| CP2/0188 | T/Ma | C02 | M/41 | T4N2bM0 | Poor | 27 | 27 | IH |
| CP2/0195 | T/Ma | C04 | M/47 | T1N1M0 | Moderate | 33 | 27 | IH |
| CP2/0196 | T/Ma | C04 | M/69 | T4N2cM0 | Well | 52 | 41 | IH |
| CP2/1002 | T/Ma | C02 | M/59 | T2N2CM0 | Well | 34 | 41 | IH |
| CP2/1003 | T | C03 | F/59 | T2N0Mx | Well | 20 | Neg | IH |
| CP2/1008 | T/Ma | C03 | F/68 | T4N1M0 | Moderate | 18 | 18 | IH |
| CP2/1010 | T/Ma | C02 | M/58 | T1N0M0 | Moderate | 40 | 20 | IH |
| CP2/1012 | T/Ma | C02 | M/55 | T3N0M0 | Well | 38 | 30 | IH |
| CP2/1019 | T/Ma | C04 | M/37 | T4N2CM0 | Well | 21 | 21 | IH |
| CP2/1021 | T | C03 | M/67 | T4N1M0 | Well | 57 | 50 | IH |
| CP2/1022 | T/Ma | C02 | M/44 | T3N0M0 | Well | 30 | 26 | IH |
| CP2/1030 | T/Ma | C02 | M/61 | T2N0 | Moderate | 47 | 42 | IH |
| CP2/1032 | T | C03 | F/57 | T4N0M0 | Moderate | Neg | Neg | IH |
| CP2/1036 | T/Ma | C02 | M/57 | T4N1M0 | Moderate | 32 | 32 | IH |
| CP2/1041 | T/Ma | C04 | M/42 | T4N2cM0 | Moderate | 33 | 33 | IH |
| CP2/1043 | T/Ma | C03 | M/69 | T4N2bM0 | Well | 50 | 46 | IH |
| CP2/1044 | T/Ma | C02 | F/51 | T2N2AM0 | Well | 31 | 1 | IH |
| CP2/1051 | T/Ma | C06 | M/56 | T3N2bM0 | Well | 43 | 30 | IH |
| CP2/1065 | T/Ma | C02 | M/59 | T2N0M0 | Moderate | 43 | 39 | IH |
| CP2/1069 | T/Ma | C02 | M/46 | T2N0M0 | Moderate | 31 | 24 | IH |
| CP2/1071 | T/Ma | C02 | M/44 | T4N2CM0 | Moderate | 29 | 21 | IH |
| CP2/1073 | T/Ma | C02 | M/58 | T2N0M0 | Moderate | 43 | 43 | IH |
| CP2/1074 | T/Ma | C02 | M/78 | T3N0M0 | Well | 50 | 50 | IH |
| CP2/1080 | T/Ma | C04 | M/64 | T2N0M0 | Well | 53 | 40 | IH |
| CP2/1099 | T/Ma | C04 | M/56 | T4N2bM0 | Well | 39 | 34 | IH |
| CP2/1104 | T/Ma | C02 | M/67 | T3N2bM0 | Well | 52 | 52 | IH |
| CP2/1109 | T/Ma | C04 | M/52 | T2N0M0 | Moderate | 36 | 18 | IH |
| CP2/1111 | T/Ma | C03 | M/42 | T3N1M0 | Moderate | 27 | 27 | IH |
| CP2/1112 | T/Ma | C04 | F/75 | T4N2bM0 | Well | Neg | Neg | IH |
| CP2/1113 | T/Ma | C04 | F/53 | T1N0M0 | Moderate | 35 | Neg | IH |
| CP2/1114 | T/Ma | C03 | M/58 | T2N1M0 | Moderate | 45 | 40 | IH |
| CP2/1120 | T/Ma | C04 | M/47 | T3N0M0 | Well | 33 | 30 | IH |
| 264713 | T/Ma | C06 | F/54 | T4N2bM0 | NA | P | P | WB |
| 272020 | T/Ma | C04 | F/67 | T3N2bM0 | NA | P | Neg | WB |
| 275629 | T | C04 | M/54 | T4N0M0 | NA | P | P | WB |
| 277479 | Ma | C04 | M/55 | T4N3M0 | NA | P | P | WB |
| 277855 | Ma | C02 | M/52 | T3N1M0 | NA | P | P | WB |
| 278241 | T/Ma | C04 | M/53 | T4N1M0 | NA | P | P | WB |
| 278354 | Ma | C02 | M/66 | T2N0M0 | NA | P | P | WB |
| 278980 | T/Ma | C04 | F/75 | T4N2BM0 | NA | P | Neg | WB |
| 279088 | T/Ma | C04 | M/58 | T2N2cMX | NA | P | Neg | WB |
| 279131 | T/Ma | C04 | M/51 | T2N0M0 | NA | P | P | WB |
| 279627 | T/Ma | C02 | M/45 | T4N1M0 | NA | P | P | WB |
| 279930 | Ma | C04 | M/66 | T2N0MX | NA | NA | NA | WB |
| 279958 | Ma | C02 | M/62 | T4N0M0 | NA | P | Neg | WB |
| 280334 | T/Ma | C02 | M/51 | T2N2AM1 | NA | P | P | WB |
| CP1/0024 | T/Ma | C04 | F/48 | T1N0M0 | Moderate | 34 | Neg | WB |
| CP1/0032 | T/Ma | C04 | M/64 | T4N2CM0 | Poor | 49 | 45 | WB |
| CP1/0039 | Ma | C02 | M/44 | T4N2BM0 | Moderate | 34 | 32 | WB |
| CP1/0055 | T/Ma | C04 | M/56 | T4N2BM0 | Well | 36 | 31 | WB |
| CP1/0057 | T/Ma | C02 | M/57 | T4N2BM0 | Moderate | 49 | 46 | WB |
| CP1/0171 | T/Ma | C04 | M/70 | T2N1M0 | Well | Neg | 59 | WB |
| CP1/0213 | T/Ma | C04 | M/68 | T4N1M0 | Well | 54 | 46 | WB |
| CP1/0233 | T/Ma | C04 | F/39 | T4N2CM0 | Poor | Neg | Neg | WB |
| CP3/0034 | T | C02 | F/51 | T2N2aM0 | Moderate | Neg | Neg | WB |
| CP3/0056 | T | C02 | M/70 | T1N0M0 | NA | 52 | 52 | WB |
| CP3/0082 | T/Ma | C02/C04 | M/59 | T4N1M0 | Well | 37 | 39 | WB |
| CP3/0114 | T | C02 | M/72 | T4N2cM0 | Poor | 50 | 34 | WB |
| CP3/0121 | T | C02 | M/50 | T4N2bM0 | Well | 34 | 32 | WB |

^a^Sites according to WHO: C02=**Malignant neoplasm of other and unspecified parts of tongue; C03=Malignant neoplasm of gum; C04=Malignant neoplasm of floor of mouth; C06=Malignant neoplasm of other and unspecified parts of mouth**
