# Supplementary material for: Proteomic Approaches Identify Members of Cofilin Pathway Involved in Oral Tumorigenesis
Source: PLoS One. 2012 Dec 5;7(12):e50517. doi: 10.1371/journal.pone.0050517 (PMC3515627; doi:10.1371/journal.pone.0050517)
Supplement: Table S2 — Pools organized into groups according to TNM system. 1-DE = one-dimensional gel electrophoresis; 2-DE = two-dimensional gel electrophoresis. (DOC) [file pone.0050517.s005.doc]

**Supporting Table 2.** **Pools organized into** groups according **to TNM system.** 1-DE=one-dimensional electrophoresis; 2-DE=two-dimensional electrophoresis.

| **Pool** | **Site^a^** | **Case** | **Type of sample** | **Group** | **Technique** |
| --- | --- | --- | --- | --- | --- |
| **1** | C02/C04 | CP2/0051 CP2/0132 CP3/0139 CP3/0280 CP3/0292 | Tumors | T1-2N+ | 1-DE |
| **2** | C02/C04 | CP1/0031 CP1/0151 CP1/0191 CP1/0277 CP1/0283 | Tumors | T3N0 | 1-DE |
| **3** | C02/C04 | CP1/0151 CP1/0262 CP1/0277 CP2/0051 CP3/0280 CP3/0292 | Surgical margins | T1-2N+ T3N0 | 1-DE |
| **4** | C02/C04 | CP1/0094 CP2/0120 CP3/0012 CP3/0046 CP3/0049 CP3/0050 CP3/0083 CP3/0087 CP3/0139 CP3/0193 | Tumors | T1-2N+ | 2-DE |
| **5** | C02/C04 | CP1/0094 CP2/0120 CP2/0175 CP3/0012 CP3/0049 CP3/0050 CP3/0083 CP3/0087 | Surgical margins | T1-2N+ | 2-DE |
| **6** | C02/C04 | CP1/0017 CP1/0075 CP1/0080 CP1/0083 CP2/0093 CP3/0004 CP3/0101 CP3/0120 CP3/0138 | Tumors | T2-3N0 | 2-DE |
| **7** | C02/C04 | CP1/0017 CP1/0051 CP1/0075 CP1/0080 CP1/0083 CP2/0185 CP2/1004 CP3/0101 CP3/0120 | Surgical margins | T2-3N0 | 2-DE |

^a^Sites according to WHO: C02=**Tongue; C04=Floor of mouth**
