# Supplementary material for: Proteomic Approaches Identify Members of Cofilin Pathway Involved in Oral Tumorigenesis
Source: PLoS One. 2012 Dec 5;7(12):e50517. doi: 10.1371/journal.pone.0050517 (PMC3515627; doi:10.1371/journal.pone.0050517)
Supplement: Table S3 — Protein identification in “more-aggressive” (MA), “less-aggressive” (LA) tumor groups and their surgical margins (SM) by 1-DE and Scaffold software according to quantitative value. Proteins with a fold change of at least 1.5 were considered with differential abundance between the groups. (PDF) [file pone.0050517.s006.pdf]

Supporting Table 3. **List of 248 proteins identified in "more-aggressive" (MA), "less-aggressive" (LA) tumors and surgical margins (SM) by 1-DE, MS and Scaffold software according to quantitative value.** Proteins with a fold change of at least 1.5 were considered with differential abundance between the categories.

			**Quantitative value**			**Fold change**		
**Gene symbol**	**Protein name**	**Accession Number**	**SM**	**LA**	**MA**	**MA/SM**	**LA/SM**	**MA/LA**
A2M	Alpha-2-macroglobulin	gi|112911 (+5)	2.63	0	1	0.4	<1.5	
ACTB	Actin, cytoplasmic 1	gi|14250401 (+8)	22.33	19.38	30.43	1.4	0.9	1.6
ACTC1	Actin, alpha cardiac muscle 1	gi|158254664 (+2)	23.64	17.69	21.87	0.9	0.7	1.2
ACTN1	Alpha-actinin-1	gi|194097350 (+6)	1	3.37	10.46	10.5	3.4	3.1
AHNAK	Neuroblast differentiation-associated protein AHNAK	gi|61743954	1	10.11	8.56	8.6	10.1	0.8
ALB	Serum albumin	gi|122920512 (+6)	288.95	190.4	173.06	0.6	0.7	0.9
ALDH3A1	Aldehyde dehydrogenase, dimeric NADP-preferring	gi|178375 (+3)	1	0.84	0	-	0.8	-
ALDOA	Fructose-bisphosphate aldolase A	gi|119600342 (+6)	1.31	0.84	0.95	0.7	0.6	1.1
ANP32A	ANP32A protein	gi|115528927 (+4)	1	1.68	1.9	1.9	1.7	1.1
ANXA1	Annexin A1	gi|119582950 (+2)	18.39	20.22	9.51	0.5	1.1	0.5
ANXA2	Annexin A2	gi|16306978 (+5)	14.45	17.69	25.67	1.8	1.2	1.5
ANXA4	Annexin A4	gi|1703319 (+5)	1	1.68	0.95	1.0	1.7	0.6
ANXA5	Annexin A5	gi|157831404 (+3)	2.63	13.48	7.61	2.9	5.1	0.6
APCS	Serum amyloid P-component	gi|337758 (+2)	6.57	0.84	0.95	0.1	0.1	1.1
APOA1	Apolipoprotein A-I	gi|90108664	13.13	7.58	7.61	0.6	0.6	1.0
ARPC2	Actin-related protein 2/3 complex subunit 2	gi|5031599	1	1.68	0.95	1.0	1.7	0.6
ASPN	Asporin	gi|13625797 (+3)	1.31	1.68	2.85	2.2	1.3	1.7
ATP1B3	Sodium/potassium transporting ATPase subunit beta-3	gi|4502281	1	0	1.9	1.9		>1.5
ATP5B	ATP synthase subunit beta, mitochondrial	gi|32189394 (+1)	2.63	6.74	13.31	5.1	2.6	2.0
BGN	Biglycan	gi|119593267 (+9)	1.31	8.42	6.66	5.1	6.4	0.8
C4B	Complement C4-B	gi|1314244 (+19)	2.63	1	1	0.4	0.4	1.0
CALM1	Calmodulin	gi|16974825	1.31	0.84	2.85	2.2	0.6	3.4
CALML5	Calmodulin-like protein 5	gi|21465435 (+1)	1	0.84	1.9	1.9	0.8	2.3
CAP1	Adenylyl cyclase-associated protein 1	gi|116241280 (+12)	1	1	1.9	1.9	1.0	1.9
CAPZA1	F-actin-capping protein subunit alpha-1	gi|158260331 (+1)	1.31	1.68	2.85	2.2	1.3	1.7
CFL1	Cofilin-1	gi|119594857 (+3)	1.31	1.68	1.9	1.5	1.3	1.1
CKM	Creatine kinase M-type or M-CK	gi|119577741 (+3)	11.82	5.05	1	0.1	0.4	0.2
CLIC1	Chloride intracellular channel protein 1	gi|14251209 (+5)	1	1.68	0.95	1.0	1.7	0.6
CLU	Clusterin	gi|178855 (+7)	1.31	1.68	0.95	0.7	1.3	0.6
COL12A1	Collagen alpha-1(XII) chain	gi|119569133 (+4)	1	1.68	1	1.0	1.7	0.6
COL1A1	Collagen alpha-1(I) chain	gi|110349772 (+6)	1.31	3.37	2.85	2.2	2.6	0.8
COL1A2	Collagen alpha-2(I) chain	gi|119597201 (+7)	0	4.21	4.75	>1.5	>1.5	1.1
COL6A1	Collagen alpha-1(VI) chain	gi|87196339	7.88	2.53	4.75	0.6	0.3	1.9
COL6A3	Collagen alpha-3(VI) chain	gi|119591511 (+9)	14.45	13.48	15.21	1.1	0.9	1.1
CPA3	Mast cell carboxypeptidase A	gi|115887 (+3)	1.31	1	1	0.8	0.8	1.0
CRYAB	Alpha-crystallin B chain	gi|227018373 (+1)	2.63	0	1.9	0.7	<1.5	>1.5
CSTA	Cystatin-A	gi|15988456 (+1)	1	3.37	1	1.0	3.4	0.3
CTSD	Cathepsin D	gi|157879202 (+1)	1.31	5.05	5.71	4.4	3.9	1.1
CTSG	Cathepsin G	gi|20664221 (+2)	1	1.68	0.95	1.0	1.7	0.6
CYB5R3	NADH-cytochrome b5 reductase 3	gi|119593674 (+9)	1	1	1.9	1.9	1.0	1.9
DCN	Decorin	gi|119617856 (+2)	3.94	4.21	2.85	0.7	1.1	0.7

DEFA1	Neutrophil defensin 1	gi|228797 (+4)	0	1.68	1		>1.5	0.6
DES	Desmin	gi|11907570 (+15)	1	5.9	9.51	9.5	5.9	1.6
DPT	Dermatopontin	gi|21708099 (+2)	1.31	0.84	0.95	0.7	0.6	1.1
DSP	Desmoplakin	gi|1147813 (+1)	1	4.21	1.9	1.9	4.2	0.5
EEF1A1	Elongation factor 1-alpha 1	gi|31092 (+6)	7.88	17.69	10.46	1.3	2.2	0.6
EEF1D	Elongation factor 1-delta	gi|10436857 (+10)	1	3.37	2.85	2.9	3.4	0.8
ENO1	Alpha-enolase	gi|203282367 (+5)	1.31	9.27	16.16	12.3	7.1	1.7
FGA	Fibrinogen alpha chain	gi|11761629 (+6)	5.25	4.21	1	0.2	0.8	0.2
FHL1	Four and a half LIM domains protein 1	gi|1381814 (+11)	2.63	1	1	0.4	0.4	1.0
FN1	Fibronectin	gi|119590941 (+17)	1	13.48	5.71	5.7	13.5	0.4
GAPDH	Glyceraldehyde-3-phosphate dehydrogenase	gi|31645	17.07	19.38	32.33	1.9	1.1	1.7
GNB2L1	Guanine nucleotide-binding protein subunit beta-2-like 1	gi|119574078 (+9)	0	2.53	0.95		>1.5	0.4
GPI	Glucose-6-phosphate isomerase or GPI	gi|14488680 (+6)	1	1	1.9	1.9	1.0	1.9
GSTP1	Glutathione S-transferase P or GSTP1-1	gi|11514451 (+15)	1	2.53	1.9	1.9	2.5	0.8
H1F0	Histone H1.0	gi|194387670 (+1)	1.31	1.68	2.85	2.2	1.3	1.7
H1FX	Histone H1x	gi|5174449	1	1.68	2.85	2.9	1.7	1.7
HBA1/HBA2	Hemoglobin subunit alpha	gi|157883730 (+3)	18.39	9.27	3.8	0.2	0.5	0.4
HBD	Hemoglobin subunit delta	gi|4504351 (+2)	38.09	24.43	1	0.0	0.6	0.0
HIST1H1B	Histone H1.5	gi|4885381	2.63	5.9	9.51	3.6	2.2	1.6
HIST1H1C	Histone H1.2	gi|4885375	13.13	11.79	21.87	1.7	0.9	1.9
HIST1H2AJ	Histone H2A type 1-J	gi|10800144 (+6)	23.64	26.12	33.28	1.4	1.1	1.3
HIST1H2B	Histone H2B type 1	gi|10800138 (+14)	19.7	27.8	31.38	1.6	1.4	1.1
HIST1H2BB	Histone H2B type 1-B	gi|10800140 (+7)	19.7	24.43	33.28	1.7	1.2	1.4
HIST1H3	Histone H3.1	gi|1568559 (+8)	3.94	5.9	4.75	1.2	1.5	0.8
HIST1H4	Histone H4	gi|119575932 (+5)	6.57	10.95	16.16	2.5	1.7	1.5
HMGB1	High mobility group protein B1	gi|114649280 (+12)	1	2.53	0	-	2.5	<1.5
HNRNPA2B1	Heterogeneous nuclear ribonucleoproteins A2/B1	gi|14043072 (+2)	2.63	10.11	5.71	2.2	3.8	0.6
HNRNPC	Heterogeneous nuclear ribonucleoproteins C1/C2	gi|109082737 (+12)	2.63	6.74	7.61	2.9	2.6	1.1
HNRNPK	Heterogeneous nuclear ribonucleoprotein K	gi|114625192 (+11)	1	4.21	4.75	4.8	4.2	1.1
HNRNPU	Heterogeneous nuclear ribonucleoprotein U	gi|14141161 (+12)	1	0.84	1	1.0	0.8	1.2
HNRPA1	Heterogeneous nuclear ribonucleoprotein A1	gi|119617173 (+5)	0	2.53	1.9	>1.5	>1.5	0.8
HP	Haptoglobin	gi|119579598 (+1)	10.51	8.42	2.85	0.3	0.8	0.3
HPX	Hemopexin	gi|11321561 (+4)	1.31	1	1	0.8	0.8	1.0
HSP90AA1	Heat shock protein HSP 90-alpha	gi|153792590 (+4)	1	1	8.56	8.6	1.0	8.6
HSP90AB1	HSP90AB1 protein	gi|194386896 (+3)	2.63	9.27	9.51	3.6	3.5	1.0
HSP90B1	Endoplasmin	gi|15010550 (+2)	1	2.53	3.8	3.8	2.5	1.5
HSPA8	Heat shock cognate 71 kDa protein	gi|193788318 (+6)	1	1.68	1.9	1.9	1.7	1.1
HSPB1	Heat shock protein beta-1 or HSP 27	gi|4504517 (+1)	5.25	10.95	10.46	2.0	2.1	1.0
HSPD1	60 kDa heat shock protein, mitochondrial or HSP-60	gi|189502784 (+5)	1	2.53	4.75	4.8	2.5	1.9
HYOU1	HYOU1 protein	gi|116283339 (+6)	1	1.68	0	-	1.7	<1.5
IGHA1	Ig alpha-1 chain C region	gi|113584 (+31)	1.31	4.21	1	0.8	3.2	0.2
IGHG3	Ig gamma-3 chain C region	gi|10334541 (+2)	15.76	12.64	1	0.1	0.8	0.1
IGLC7	Ig lambda-7 chain C region	gi|1335160 (+6)	6.57	10.95	1	0.2	1.7	0.1
KRT1	Keratin, type II cytoskeletal 1	gi|11935049 (+1)	11.82	11.79	5.71	0.5	1.0	0.5
KRT10	Keratin, type I cytoskeletal 10	gi|119581085 (+4)	2.63	5.05	7.61	2.9	1.9	1.5
KRT13	Keratin, type I cytoskeletal 13	gi|131412225	36.78	32.01	36.13	1.0	0.9	1.1

KRT14	Keratin, type I cytoskeletal 14	gi|12803709 (+2)	14.45	53.08	67.51	4.7	3.7	1.3
KRT16	Keratin, type I cytoskeletal 16	gi|1195531 (+1)	1	35.38	45.64	45.6	35.4	1.3
KRT17	Keratin, type I cytoskeletal 17	gi|21754583 (+1)	1	16.01	39.94	39.9	16.0	2.5
KRT19	Keratin, type I cytoskeletal 19	gi|24234699 (+2)	11.82	21.06	30.43	2.6	1.8	1.4
KRT4	Keratin, type II cytoskeletal 4	gi|109255249 (+5)	32.84	5.9	6.66	0.2	0.2	1.1
KRT5	Keratin, type II cytoskeletal 5	gi|119395754 (+1)	34.15	80.88	67.51	2.0	2.4	0.8
KRT6A	Keratin, type II cytoskeletal 6A	gi|114644568 (+4)	36.78	76.67	70.36	1.9	2.1	0.9
KRT8	Keratin, type II cytoskeletal 8	gi|119617057 (+5)	1	3.37	11.41	11.4	3.4	3.4
KRT9	Keratin, type I cytoskeletal 9	gi|435476 (+1)	1.31	3.37	1	0.8	2.6	0.3
LAMP2	Lysosomal-associated membrane protein 2C	gi|169790833 (+5)	1	1	0.95	1.0	1.0	1.0
LCN2	Neutrophil gelatinase-associated lipocalin	gi|119608154 (+17)	1	1.68	1	1.0	1.7	0.6
LDB3	LIM domain-binding protein 3	gi|119600728 (+7)	2.63	1	1	0.4	0.4	1.0
LGALS1	Galectin-1	gi|42542977 (+2)	1	5.05	6.66	6.7	5.1	1.3
LGALS7	Galectin-7	gi|34783544 (+2)	1.31	2.53	0.95	0.7	1.9	0.4
LMNA	Prelamin-A/C	gi|119573381 (+13)	1	4.21	3.8	3.8	4.2	0.9
LUM	Lumican	gi|4505047 (+1)	10.51	22.75	17.12	1.6	2.2	0.8
MANF	Mesencephalic astrocyte-derived neurotrophic factor	gi|119585542 (+5)	1	0.84	1.9	1.9	0.8	2.3
MB	Myoglobin	gi|119580469 (+6)	6.57	0	2.85	0.4	<1.5	>1.5
MDH2	Malate dehydrogenase, mitochondrial	gi|21735621 (+5)	2.63	3.37	0	<1.5	1.3	<1.5
MYH2	Myosin-2	gi|153791586 (+1)	39.4	0.84	7.61	0.2	0.0	9.1
MYH7	Myosin-7	gi|115496169 (+4)	22.33	1	11.41	0.5	0.0	11.4
MYH9	Myosin-9	gi|12667788	1.31	5.05	14.26	10.9	3.9	2.8
MYL1	Myosin light chain 1/3, skeletal muscle isoform or MLC1/MLC3	gi|119590891 (+4)	6.57	1	0.95	0.1	0.2	1.0
MYL12B	Myosin regulatory light chain 12B	gi|15809016 (+3)	2.63	7.58	6.66	2.5	2.9	0.9
MYL2	Myosin regulatory light chain 2, ventricular/cardiac muscle isoform or MLC-2v	gi|119618350 (+3)	3.94	1	0.95	0.2	0.3	1.0
MYL6	Myosin light polypeptide 6	gi|113812151 (+5)	1.31	4.21	3.8	2.9	3.2	0.9
MYLPF	Myosin regulatory light chain 2, skeletal muscle isoform or MLC2B	gi|1220346 (+2)	7.88	1	1.9	0.2	0.1	1.9
MYOZ1	Myozenin-1	gi|10864053 (+1)	3.94	1	1	0.3	0.3	1.0
NACA	Nascent polypeptide-associated complex subunit alpha	gi|163965366 (+3)	1.31	3.37	2.85	2.2	2.6	0.8
NME2	Nucleoside diphosphate kinase B	gi|66392203 (+1)	1	2.53	0.95	1.0	2.5	0.4
NPM1	Nucleophosmin	gi|10835063 (+8)	1.31	0.84	2.85	2.2	0.6	3.4
NRP1	Neuropilin-1	gi|160877748	15.76	14.32	1	0.1	0.9	0.1
OGN	Mimecan	gi|194380046 (+2)	6.57	1.68	2.85	0.4	0.3	1.7
ORM1	Alpha-1-acid glycoprotein 1	gi|112877 (+3)	2.63	0.84	0.95	0.4	0.3	1.1
P4HB	Protein disulfide-isomerase	gi|194388618 (+2)	1	3.37	2.85	2.9	3.4	0.8
PACAP	Plasma cell-induced resident endoplasmic reticulum protein	gi|117938314	1	1.68	0.95	1.0	1.7	0.6
PDIA3	Protein disulfide-isomerase A3	gi|114656687 (+8)	1	0.84	1.9	1.9	0.8	2.3
PDIA6	Protein disulfide-isomerase A6	gi|1710248 (+5)	1	4.21	8.56	8.6	4.2	2.0
PEBP1	Phosphatidylethanolamine-binding protein 1	gi|194387966 (+2)	1.31	1.68	2.85	2.2	1.3	1.7
PFDN2	Prefoldin subunit 2	gi|12408675	1	0.84	1.9	1.9	0.8	2.3
PFN1	Profilin-1	gi|157833469 (+3)	1	2.53	2.85	2.9	2.5	1.1
PHB2	Prohibitin-2	gi|119609105 (+4)	1	0	1.9	1.9		>1.5
POSTN	Periostin	gi|166343765 (+13)	1	6.74	14.26	14.3	6.7	2.1
PPIA	Peptidyl-prolyl cis-trans isomerase A or PPIase A or Cyclophilin A	gi|10863927 (+3)	2.63	10.11	9.51	3.6	3.8	0.9
PPIB	Peptidyl-prolyl cis-trans isomerase B	gi|1310882 (+4)	2.63	8.42	11.41	4.3	3.2	1.4
PRDX1	Peroxiredoxin-1	gi|119627382 (+3)	7.88	5.9	4.75	0.6	0.7	0.8

PRDX2	Peroxiredoxin-2	gi|194375974 (+2)	7.88	5.05	0.95	0.1	0.6	0.2
PRKCSH	Glucosidase 2 subunit beta	gi|119604622 (+6)	1	1	2.85	2.9	1.0	2.9
PSAP	Proactivator polypeptide	gi|110224476 (+16)	1	0.84	1.9	1.9	0.8	2.3
PSMA1	Proteasome subunit alpha type-1	gi|119588883 (+4)	1	0.84	0.95	1.0	0.8	1.1
PSME1	Proteasome activator complex subunit 1	gi|119586510 (+3)	1	0.84	1.9	1.9	0.8	2.3
RAP1A	Ras-related protein Rap-1A	gi|14595132	1	1.68	1.9	1.9	1.7	1.1
RPL11	60S ribosomal protein L11	gi|13385408 (+4)	1.31	2.53	1.9	1.5	1.9	0.8
RPL12	60S ribosomal protein L12	gi|119608073 (+3)	0	2.53	1.9	>1.5	>1.5	0.8
RPL13	60S ribosomal protein L13	gi|15431295 (+2)	1	1.68	1.9	1.9	1.7	1.1
RPL18	60S ribosomal protein L18	gi|119572744 (+4)	1	3.37	2.85	2.9	3.4	0.8
RPL19	60S ribosomal protein L19	gi|119580972 (+5)	1	0.84	1.9	1.9	0.8	2.3
RPL22	60S ribosomal protein L22	gi|108996192 (+4)	1.31	3.37	0.95	0.7	2.6	0.3
RPL23	60S ribosomal protein L23	gi|13097600 (+2)	1	0.84	1	1.0	0.8	1.2
RPL23A	60S ribosomal protein L23a	gi|119571516 (+5)	1.31	3.37	5.71	4.4	2.6	1.7
RPL24	60S ribosomal protein L24	gi|119600188 (+3)	1.31	2.53	2.85	2.2	1.9	1.1
RPL26	60S ribosomal protein L26	gi|292435 (+2)	0	2.53	3.8	>1.5	>1.5	1.5
RPL27	60S ribosomal protein L27	gi|119581314 (+2)	1	0.84	0.95	1.0	0.8	1.1
RPL28	60S ribosomal protein L28	gi|13904866 (+4)	1	2.53	2.85	2.9	2.5	1.1
RPL31	60S ribosomal protein L31	gi|148746199 (+6)	1.31	2.53	2.85	2.2	1.9	1.1
RPL34	60S ribosomal protein L34	gi|1008856 (+1)	1	0	0.95	1.0		
RPL4	60S ribosomal protein L4	gi|119598179 (+5)	1	0.84	0.95	1.0	0.8	1.1
RPL5	60S ribosomal protein L5	gi|187609312 (+5)	1	1.68	0.95	1.0	1.7	0.6
RPL6	60S ribosomal protein L6	gi|16753227 (+5)	1	4.21	3.8	3.8	4.2	0.9
RPL8	60S ribosomal protein L8	gi|119602459 (+2)	1	1.68	1.9	1.9	1.7	1.1
RPLP0	60S acidic ribosomal protein P0	gi|119618576 (+6)	1	3.37	5.71	5.7	3.4	1.7
RPLP2	60S acidic ribosomal protein P2	gi|4506671	1.31	5.05	5.71	4.4	3.9	1.1
RPS10	40S ribosomal protein S10	gi|119624187 (+3)	1	0.84	1.9	1.9	0.8	2.3
RPS11	40S ribosomal protein S11	gi|4506681	1	1.68	0.95	1.0	1.7	0.6
RPS13	40S ribosomal protein S13	gi|4506685	1	0.84	1.9	1.9	0.8	2.3
RPS14	40S ribosomal protein S14	gi|5032051	0	1.68	1.9	>1.5	>1.5	1.1
RPS16	40S ribosomal protein S16	gi|109124686 (+3)	1.31	3.37	1.9	1.5	2.6	0.6
RPS18	40S ribosomal protein S18	gi|114606879 (+1)	1	4.21	6.66	6.7	4.2	1.6
RPS19	40S ribosomal protein S19	gi|119577478 (+2)	0	3.37	6.66	>1.5	>1.5	2.0
RPS23	40S ribosomal protein S23	gi|4506701	1.31	1.68	0.95	0.7	1.3	0.6
RPS4X	40S ribosomal protein S4, X isoform	gi|119592221 (+5)	1.31	0.84	0.95	0.7	0.6	1.1
RPS5	40S ribosomal protein S5	gi|119592989 (+3)	1	0.84	0.95	1.0	0.8	1.1
RPS6	40S ribosomal protein S6	gi|109111443 (+7)	1.31	2.53	1.9	1.5	1.9	0.8
RPS7	40S ribosomal protein S7	gi|337518 (+1)	0	4.21	0.95		>1.5	0.2
RPS8	40S ribosomal protein S8	gi|119627428 (+2)	1	2.53	2.85	2.9	2.5	1.1
RPS9	40S ribosomal protein S9	gi|14141193 (+1)	1	2.53	2.85	2.9	2.5	1.1
RSU1	Ras suppressor protein 1	gi|34577083 (+2)	1	1.68	0.95	1.0	1.7	0.6
S100A6	Protein S100-A6 or calcyclin	gi|7657532	2.63	1.68	0.95	0.4	0.6	0.6
S100A7	Protein S100-A7 or psoriasin	gi|115298657 (+3)	1	2.53	0.95	1.0	2.5	0.4
S100A8	Protein S100-A8	gi|21614544 (+2)	6.57	6.74	6.66	1.0	1.0	1.0
S100A9	Protein S100-A9 or Calgranulin-B	gi|189053201 (+3)	6.57	20.22	10.46	1.6	3.1	0.5
SEPT2	Septin-2	gi|1040689 (+12)	1	1	1.9	1.9	1.0	1.9

SERPINA1	Alpha-1-antitrypsin	gi|110350939 (+17)	5.25	10.95	5.71	1.1	2.1	0.5
SERPINC1	Antithrombin-III	gi|179161 (+3)	1	1.68	0	-	1.7	<1.5
SERPINH1	Serpin H1	gi|119595384 (+6)	1	0.84	2.85	2.9	0.8	3.4
SET	Protein SET	gi|119608226 (+9)	1	0.84	0.95	1.0	0.8	1.1
SFN	14-3-3 protein sigma or stratifin	gi|16306737 (+2)	2.63	11.79	11.41	4.3	4.5	1.0
SH3BGRL	SH3 domain-binding glutamic acid-rich-like protein	gi|12052955 (+3)	1	1	0.84	0.8	1.0	0.8
SOD2	Superoxide dismutase [Mn], mitochondrial	gi|110590806 (+26)	1.31	2.53	1.9	1.5	1.9	0.8
SPRR3	Small proline-rich protein 3	gi|4885607 (+3)	1.31	0.84	1	0.8	0.6	1.2
SRSF1	Serine/arginine-rich splicing factor 1	gi|118582269 (+5)	1	2.53	0.95	1.0	2.5	0.4
SRSF3	Serine/arginine-rich splicing factor 3	gi|119624305 (+4)	1	1.68	2.85	2.9	1.7	1.7
SSR1	Translocon-associated protein subunit alpha	gi|109069546 (+8)	1	0.84	0	-	0.8	-
STMN1	Stathmin	gi|197692339 (+2)	1	0.84	2.85	2.9	0.8	3.4
SUB1	Activated RNA polymerase II transcriptional coactivator p15	gi|16307067 (+3)	1	1.68	1.9	1.9	1.7	1.1
TAGLN	Transgelin	gi|119587704 (+3)	1	0.84	4.75	4.8	0.8	5.7
TAGLN2	Transgelin-2	gi|12803567 (+3)	1	6.74	3.8	3.8	6.7	0.6
TALDO1	Transaldolase	gi|48257056 (+1)	1	2.53	0.95	1.0	2.5	0.4
TF	Serotransferrin	gi|110590599 (+4)	7.88	3.37	2.85	0.4	0.4	0.8
THY1	Thy-1 membrane glycoprotein	gi|119587893 (+3)	1	2.53	0	-	2.5	<1.5
TMED9	Transmembrane emp24 domain-containing protein 9	gi|119605373 (+4)	1	0	0.95	1.0		
TNC	Tenascin	gi|119607840 (+5)	1	5.9	15.21	15.2	5.9	2.6
TNNT3	Troponin T, fast skeletal muscle	gi|112789538 (+5)	5.25	1	0.95	0.2	0.2	1.0
TPI1	Triosephosphate isomerase 1 or TIM	gi|17389815 (+6)	1.31	2.53	5.71	4.4	1.9	2.3
TPM1	Tropomyosin alpha-1 chain	gi|49660014 (+1)	14.45	8.42	9.51	0.7	0.6	1.1
TPM2	Tropomyosin beta chain	gi|42476296	13.13	9.27	7.61	0.6	0.7	0.8
TPM3	Tropomyosin alpha-3 chain	gi|114155140 (+1)	10.51	8.42	7.61	0.7	0.8	0.9
TPM3	Tropomyosin alpha-3 chain	gi|197099510	10.51	9.27	8.56	0.8	0.9	0.9
TPM3	Tropomyosin alpha-3 chain	gi|114155144	1	1	8.56	8.6	1.0	8.6
TPM4	Tropomyosin alpha-4 chain	gi|4507651	1	10.95	7.61	7.6	11.0	0.7
TUBA1C	Tubulin alpha-1C chain	gi|14389309 (+6)	7.88	10.95	12.36	1.6	1.4	1.1
TUBB	Tubulin beta chain	gi|18088719 (+3)	0	11.79	15.21	>1.5	>1.5	1.3
UBC	Polyubiquitin-C	gi|2627129 (+29)	5.25	9.27	11.41	2.2	1.8	1.2
VDAC1	Voltage-dependent anion-selective channel protein 1	gi|198443050 (+4)	1	1.68	3.8	3.8	1.7	2.3
VDAC2	Similar to Voltage-dependent anion-selective channel	gi|113412878 (+12)	1	3.37	0.95	1.0	3.4	0.3
VIM	Vimentin	gi|62414289	26.27	40.44	47.54	1.8	1.5	1.2
YWHAB	14-3-3 protein beta/alpha	gi|4507949 (+1)	1	9.27	10.46	10.5	9.3	1.1
YWHAE	14-3-3 protein epsilon	gi|114665591 (+2)	2.63	9.27	11.41	4.3	3.5	1.2
YWHAG	14-3-3 protein gamma	gi|193785841 (+4)	1	8.42	8.56	8.6	8.4	1.0
		gi|4929993	57.79	27.8	1	0.0	0.5	0.0
		gi|166007160	28.9	26.12	9.51	0.3	0.9	0.4
		gi|1065111 (+11)	2.63	1.68	0.95	0.4	0.6	0.6
		gi|21669601	5.25	9.27	1.9	0.4	1.8	0.2
		gi|16554039	18.39	24.43	6.66	0.4	1.3	0.3
		gi|229959 (+1)	77.49	34.54	28.53	0.4	0.4	0.8
		gi|194376242	17.07	14.32	6.66	0.4	0.8	0.5
		gi|14719797 (+4)	9.19	5.05	3.8	0.4	0.5	0.8
		gi|218681905 (+1)	15.76	15.16	6.66	0.4	1.0	0.4

	gi|229601	26.27	30.33	11.41	0.4	1.2	0.4
	gi|119389654 (+17)	1.31	0.84	1	0.8	0.6	1.2
	gi|119602344 (+7)	1.31	1	1	0.8	0.8	1.0
	gi|119613805 (+2)	1	0	0.95	1.0		
	gi|1096067 (+5)	1	1.68	0.95	1.0	1.7	0.6
	gi|114647215 (+16)	1	0.84	1	1.0	0.8	1.2
	gi|119573745 (+3)	1	1.68	1	1.0	1.7	0.6
	gi|119579121 (+5)	1	1.68	1	1.0	1.7	0.6
	gi|1065033 (+140)	1	0.84	1	1.0	0.8	1.2
	gi|13786847 (+2)	1.31	5.05	1.9	1.5	3.9	0.4
	gi|119607495 (+1)	1	2.53	1.9	1.9	2.5	0.8
	gi|119619436 (+1)	1	0.84	1.9	1.9	0.8	2.3
	gi|17471847 (+1)	1	1	1.9	1.9	1.0	1.9
	gi|119582155 (+5)	2.63	3.37	5.71	2.2	1.3	1.7
	gi|119569329 (+1)	2.63	4.21	5.71	2.2	1.6	1.4
	gi|119568094 (+5)	1.31	3.37	2.85	2.2	2.6	0.8
	gi|230867	3.94	5.05	11.41	2.9	1.3	2.3
	gi|13786849 (+3)	1.31	5.9	4.75	3.6	4.5	0.8
	gi|1065361 (+6)	1	2.53	3.8	3.8	2.5	1.5
	gi|119601423 (+7)	1.31	3.37	5.71	4.4	2.6	1.7
	gi|161172138 (+5)	2.63	14.32	13.31	5.1	5.4	0.9
	gi|119597983 (+10)	0	1.68	2.85	>1.5	>1.5	1.7
	gi|11276938 (+4)	0	2.53	0.95		>1.5	0.4
