# Supplementary material for: Proteomic Approaches Identify Members of Cofilin Pathway Involved in Oral Tumorigenesis
Source: PLoS One. 2012 Dec 5;7(12):e50517. doi: 10.1371/journal.pone.0050517 (PMC3515627; doi:10.1371/journal.pone.0050517)
Supplement: Table S4 — Differentially expressed proteins identified by 2-DE followed by mass spectrometry analysis in “more-aggressive” (MA), “less-aggressive” (LA) tumor groups and their surgical margins (SM). MA/SM, LA/SM and MA/LA abundance ratio. (DOC) [file pone.0050517.s007.doc]

**Supporting Table 4. Differentially expressed proteins identified by 2-DE followed by mass spectrometry analysis in "more-aggressive" (MA), "less-aggressive" (LA) tumors and surgical margins (SM).** MA/SM, LA/SM and MA/LA abundance ratio.

| **Protein** | Accession number | Theoretical mass kDa/p*I* | **# matched peptides** | **Mascot score^a^** | **MA/SM** | **LA/SM** | **MA/LA** |
| --- | --- | --- | --- | --- | --- | --- | --- |
| Actin, cytoplasmic 2 | P63261 | 41.792/5.31 | 4 | 123 | 0.5045* | 0.9482 | 0.9650 |
| **Alpha-enolase** | P06733 | 47.037/6.99 | 3 | 187 | 3.9205* | 2.4116* | 2.0364* |
| Annexin A1 | P04083 | 38.583/6.64 | 3 | 131 | 0.6850 | 0.5519* | 1.1058 |
| Annexin A2 | P07355 | 38.472/7.56 | 12 | 119 | 0.3584* | 0.2892* | 2.2628* |
| Carbonic anhydrase 3 | P07451 | 29.426/6.94 | 2 | 74 | 0.2199 | 0.3928* | 2.6328* |
| Cofilin-1 | P23528 | 18.371/8.26 | 2 | 163 | 3.0768* | 2.0726* | 1.9257* |
| **Creatine kinase M-type or M-CK** | P06732 | 43.101/6.77 | 5 | 215 | 0.1841* | 0.2081* | 1.3739 |
| Galectin-7 | P47929 | 14.943/7.00 | 4 | 274 | 1.3607 | 3.2045* | 1.1674 |
| Glutathione S-transferase P or **GSTP1-1** | P09211 | 23.224/5.44 | 3 | 225 | 2.0257* | 1.3811* | 1.8671* |
| Heat shock protein β-1 or HSP 27 | P04792 | 22.782/5.98 | 4 | 233 | 2.4502* | 2.4802* | 1.2403* |
| Hemoglobin subunit β | P68871 | 15.867/6.81 | 4 | 211 | 0.5879* | 0.9061 | 1.4205* |
| Keratin, type I cytoskeletal 19 | P08727 | 44.091/5.05 | 3 | 101 | 1.9323* | 1.6344* | 0.6650* |
| Keratin, type II cytoskeletal 4 | P19013 | 57.285/6.25 | 4 | 177 | 0.5010 | 0.7408* | 0.7911 |
| Myoglobin | P02144 | 17.052/7.29 | 2 | 187 | 0.3596* | 0.2108* | 1.7494* |
| Myosin light chain 1/3, skeletal muscle isoform | P05976 | 21.013/4.97 | 4 | 222 | 0.3525* | 0.2869* | 1.1191 |
| **Myosin light chain 3** | P08590 | 21.800/5.03 | 5 | 174 | 0.2374* | 0.1789* | 2.6983* |
| Myosin regulatory light chain 2, skeletal muscle isoform | Q96A32 | 18.883/4.91 | 3 | 86 | 0.4714* | 0.2979* | 0.9345 |
| Myosin regulatory light chain 2, ventricular/cardiac muscle isoform | P10916 | 18.658/4.92 | 2 | 56 | 0.2012* | 0.3545* | 1.0044 |
| Peptidyl-prolyl cis-trans isomerase A or Cyclophilin A | P62937 | 17.881/7.82 | 1 | 107 | 2.5125* | 2.2657* | 1.4113 |
| Protein S100-A9 or Calgranulin-B | P06702 | 13.110/5.71 | 2 | 156 | 2.7548* | 2.3885* | 1.1376 |
| Serum albumin | P02768 | 66.472/5.67 | 2 | 80 | 3.6482 | 0.1846* | 3.0168 |
| **Stratifin or 14-3-3 protein sigma** | P31947 | 27.774/4.68 | 7 | 66 | 2.1116* | 3.2639* | 0.4625* |
| **Superoxide dismutase [Cu-Zn]** | P00441 | 15.804/5.70 | 2 | 102 | 3.8455* | 1.7524* | 2.1942* |
| Tropomyosin alpha-1 chain | P09493 | 32.708/4.69 | 2 | 38 | 0.5060 | 0.4820* | 1.3156 |
| Tropomyosin beta chain | P07951 | 32.850/4.66 | 3 | 146 | 0.2510* | 0.2007* | 1.6344 |
| Tropomyosin alpha-3 chain | P06753 | 32.818/4.68 | 4 | 165 | 0.4982* | 0.4041* | 0.7772 |
| Tropomyosin alpha-4 chain | P67936 | 28.390/4.67 | 3 | 107 | 3.9045* | 2.1951* | 0.6928 |
| **Troponin T, slow skeletal muscle** | P13805 | 32.816/5.86 | 3 | 127 | 1.0490 | 0.1711* | 1.4034 |
| Vimentin | P08670 | 53.520/5.06 | 4 | 150 | 2.0251* | 2.1760* | 0.7970 |

^a^Total MASCOT score: sum of individual matched peptide scores

* indicates significant differences between the sample groups (Student's t test p<0.05)
